# Manufacturer’s Encroachment and Carbon Emission Reduction Decisions Considering Cap-and-Trade Regulation and Consumers’ Low-Carbon Preference

**DOI:** 10.3390/ijerph191610407

**Published:** 2022-08-21

**Authors:** Fan Ding, Zhangping Lu, Mengfan Jin, Licheng Sun

**Affiliations:** 1 Management School, Jiangsu University, Zhenjiang 212013, China; 2 Instrument of Science & Technology Information, Jiangsu University, Zhenjiang 212013, China; 3 Department of Architecture, Xi’an Jiaotong-Liverpool University, Suzhou 215127, China

**Keywords:** manufacturer’s encroachment decision, cap-and-trade regulation, carbon emission reduction, consumers’ low-carbon preference

## Abstract

Carbon emission reduction and achieving carbon neutrality has become an inevitable trend in the sustainable development era. We investigate the manufacturer’s encroachment and carbon emission reduction decisions considering government cap-and-trade regulations and consumers’ low-carbon preference. The equilibrium decisions for the four scenarios are analytically obtained and compared based using the Stackelberg game. A comparison with and without cap-and-trade regulation under two encroachment decisions regarding member’s profits and carbon emission reduction levels are conducted. It is shown that the encroachment decision is always advantageous for the manufacturer if the government decides not to implement cap-and-trade regulation, and the retailer always loses profit. Moreover, if the carbon quota is sufficient, cap-and-trade regulation benefits the manufacturer. Otherwise, the manufacturer’s encroachment decision depends on the appropriate initial unit amount of carbon emission and unit carbon price. The retailer’s profit may not always be hurt by the manufacturer’s encroachment with cap-and-trade regulation; unless the unit carbon price exceeds a certain threshold, a higher consumer’s low-carbon preference in the encroachment scenario reduces more carbon emissions than in the no-encroachment scenario for the manufacturer. Further, the rising platform commission rate causes the platform profit to increase first and then decrease; the platform profit will slightly decrease if both products become more substitutes.

## 1. Introduction

Carbon emissions have a significant negative impact on economic development because of environmental issues such as the greenhouse effect and global climate change [1,2]. China is the largest CO_2_ emitter in the world by ratifying the “Paris Agreement-COP21”,and has pledged to enhance low-carbon development. Manufacturing industries are responsible for around 55.06% of the total energy consumption in 2019 [3]. In 2020, the Chinese government clearly stated that it strives to achieve the “carbon peak” by 2030 and complete the dual carbon goals of “carbon neutrality” by 2060. Recently, cap-and-trade regulation has become the key emission reduction mechanism for achieving carbon peak and carbon neutralization.

Cap-and-trade regulation was first introduced by the European Union Emissions Trading Scheme (EU-ETS) in 2005 [4,5] and has gradually become one of the most successful environmental regulations for reducing carbon emissions [6,7,8]. “Cap-and-trade” means “pricing air”. The government assigns certain carbon emission quotas to manufacturers and sets the annual carbon amount that the manufacturer can release. If the quota is not used up, the remaining part can be sold through the carbon trading market. On the contrary, extra parts should be purchased in the carbon trading market if exceeding the quota. In China, the Environment and Energy Exchange for carbon emission trading was launched in Shanghai, and carbon trading markets were developed in Chongqing, Tianjin, Beijing, and Guangdong [9]. Other countries have also legislated the cap-and-trade regulation, including the United States, India, and the Netherlands [10]. Tesla sells carbon quotas to rival automakers who are required to comply with emissions standards [11]. According to the Quarter report, Tesla made $679 million in the first quarter from the sale of carbon quotas, which soared from $518 million a year earlier. Tesla’s net profit can still be lost if this profit is not included.

Additionally, online purchasing has rapidly increased during the past few decades. Manufacturers are not only in charge of producing national brand (NB) products for their retailers but also have a chance to open a direct channel to sell factory brands products (FB) and compete with their retailers, which is called “manufacturer encroachment” [12,13]. Note that manufacturers produce the FB products with the same material in the same production line; the only distinction between the two products is the brand. For instance, the Chinese online retailing platform Pinduoduo launched the New Brand Initiatives project in 2018 [14], which aims to encourage Chinese manufacturers to satisfy online consumers’ demand with cost-effective products. Within about one year, hundreds of FB products emerged on the Pinduoduo platform with high sales. Thus, the encroached manufacturer can launch FB products through Pinduoduo and compete with the retailer who sells NB products through the traditional retailing channel. If ill-managed, however, the encroachment strategy may hurt the relationship between supply chain members [15].

Moreover, carbon emission reduction increases consumers’ low-carbon awareness. Rational consumers are gradually favoring environmentally friendly products. According to a Wall Street Journal poll, approximately 37% of consumers in the United States and 23% in Europe are willing to pay more than a 5% cost for environmental-friendly products [16]. Additionally, one-third of consumers will consider the environmental impact of their products while purchasing [17]. The Pinduoduo Annual Report also mentioned that consumers who prefer energy-saving products have increased by 35% [14]. Thus, it is significant for manufacturers to implement low-carbon technologies to reduce carbon emissions that consider consumers’ low-carbon preferences. Furthermore, the carbon emission reduction levels may not be the same in the encroachment and no-encroachment scenarios, and this situation will become more complicated under different government regulations. It would be a special issue when the manufacturers make carbon emission decisions considering both the encroachment decisions and cap-and-trade regulation policy.

Recently, seven carbon trading pilots have been established in China [18]. However, the cap-and-trade regulation policy currently aims at power enterprises with high carbon emissions. Thousands of small and medium-sized enterprises (SME) have not fully implemented this regulation. Besides, some issues in the carbon market, such as the carbon quota allocation, carbon pricing mechanism, and policy systems, still exist [19]. Additionally, due to the government’s low-carbon policies and consumers’ low-carbon preference, manufacturers must reach the trade-off between environmental and economic performance during production [20]. Note that the carbon emission regulation may affect the manufacturers’ operation decisions, especially the encroachment decision. For example, if the regulation tends to be stricter, the carbon trading price will increase, which results in a higher carbon emission reduction cost for manufacturers.

We are motivated by the factors mentioned above to investigate manufacturer encroaching decisions considering cap-and-trade regulation and consumers’ low-carbon preference. The manufacturer’s encroachment decisions encouraged by online platforms, taking government cap-and-trade regulation into account, have a theoretical space in the literature. The analysis above may lead to the following research questions:

**Research Question 1:** Do the cap-and-trade regulations influence manufacturer’s encroachment and optimal carbon emission reduction decisions?

**Research Question 2:** Do supply chain members benefit or suffer from the manufacturer’s encroachment considering cap-and-trade regulation?

**Research Question 3:** What impact do consumers’ low-carbon preferences have on the manufacturer’s carbon emission reduction level, supply chain members’ operation decisions, and equilibrium profits? What managerial insights can be obtained for the policy maker and firms?

The problems we investigate are fresh when examining the introduction of cap-and-trade regulation to the manufacturer’s encroachment decisions while considering the influence of consumers’ low-carbon preference, channel preference, and two substituted products. We compare four possible scenarios: laissez-faire (unregulated markets) with no encroachment (NU), laissez-faire with encroachment (EU), cap-and-trade regulation with no-encroachment (NR), and cap-and-trade regulation with encroachment (ER). There are two reasons why it is essential to investigate the unregulated marketplaces. Firstly, it is crucial to consider both the traditional scenario with no carbon emission regulation and the regulated scenario when examining the impact of the cap-and-trade regulation on manufacturers’ encroachment and chain members’ profits. Various environmental rules result in different costs and additional benefits for supply chain participants [21]. Secondly, cap-and-trade regulation is currently aimed at high carbon emission companies. For SMEs, cap-and-trade regulation has not been popularized. The results of this research may help manufacturer managers to optimize their profits and encroachment strategies, and cap-and-trade regulation can be well-implemented by the government.

The remaining sections of the paper are structured as follows. In Section 2, relevant literature is discussed. Section 3 describes the problems and models in detail. Then, we give four different models and corresponding equilibrium solutions and comparisons in Section 4. Section 5 presents numerical solutions for the equilibrium results. Discussion, Conclusions and some directions for future research are provided in Section 6 and Section 7. Finally, all equilibrium results and proofs are shown in the Supplementary Materials to make the paper more readable.

## 2. Literature Review

Related literature can be divided into two categories: supply chain management under cap-and-trade regulation and the manufacturer encroachment issue. To highlight our contribution, we review only the representative and particularly relevant literature to our study.

### 2.1. Supply Chain Management under the Cap-and-Trade Regulation

Supply chain management under the cap-and-trade regulation is an important topic in the carbon neutrality era; it is also related to our research. The existing literature on supply chain management under the cap-and-trade regulation contains research on pricing, channel selection, and carbon emission reduction decisions. The cap-and-trade model in supply chain management formed the central focus of a study by Benjaafar et al. [6]. The author initially found the importance of operating models in evaluating the effect of different environmental policies and the benefits of the investment in various carbon-efficient technologies. Drake et al. [5] studied the impact of cap-and-trade regulation and emissions tax on a firm’s technology choice and capacity decisions, and their research aimed to present the comparison between different low-carbon policies. 

Differently, we focus on the cap-and-trade regulation in the manufacturer encroachment models. Under cap-and-trade regulation, Yang et al. [22] jointly studied the manufacturer’s optimal channel selection and carbon emission reduction decision. Our study investigates the manufacturer’s encroachment strategies with and without cap-and-trade regulation, and we also consider the impact of the online platform commission rate. Anand et al. [1] argued that the competition between two firms plays a crucial role in determining the economic effects of carbon regulation. They compared a laissez-faire case with a well-chosen regulation case and proved that the well-chosen regulation could improve consumer surplus. Our research also compares unregulated market and cap-and-trade regulation. However, we focus on the issue of manufacturer’s encroachment in the dual-channel scenarios. Additionally, Liu et al. [23] explore how cap-and-trade regulation affects an online retailing selection between reseller and marketplace and supply chain member’s response. Similarly, they considered a supply chain composed of an online platform and a manufacturer. Nevertheless, we focus on implementing cap-and-trade regulation into the manufacturer’s encroachment model with two substituted products. Ji et al. [24] utilized a two-stage Stackelberg game to explore the manufacturer’s production decision and the government’s cap-setting problems. They found that the manufacturer’s profit initially increases and then decreases with the allocated cap to some extent. This result is unique and different from other literature. Additionally, Xu et al. [18] investigated the optimal manufacturer’s production and delivery time decisions and the government’s two different cap-and-trade regulation region cap setting decisions. Yu et al. [25] reported how the government’s carbon emission regulation decisions between cap-and-trade and carbon tax regulation impact a manufacturer’s product decision, which sells its product through offline and online channels.

### 2.2. Manufacturer’s Encroachment Decision

This research is related to the dual-channel supply chain literature because we also investigate the manufacturer encroachment issue. The dual-channel supply chain is the subject of current research, which largely focuses on pricing strategy, channel competition, and channel selection. In the dual-channel scenario, manufacturers fulfill the role of both a supplier and a direct rival of the retailer by selling products directly to consumers. Chiang et al. [26] showed that when consumers weakly prefer the direct channel over the retailer channel, the manufacturer’s threat of launching its direct channel could lead to a double-win situation for both retailing and online channels. Likewise, Arya et al. [27] showed that the manufacturer who has the selling cost disadvantage relative to the retailer may be motivated to decrease the wholesale price to keep the retailer channel’s demand. Furthermore, if the selling cost difference is large, the retailer may get profit from the manufacturer’s encroachment. Cai [28] supported the idea that a win–win situation develops when the retailer has a considerable advantage in base demand or operating costs. In contrast to Cai [28], Chen et al. [12] showed that all supply chain participants may favor the manufacturer’s encroachment strategy without downstream integration. Nevertheless, the no-encroachment decision could only be preferred when the OEM and the retailer are both in a single entity.

There is no doubt that introducing the online direct channel to the traditional retail channel is an effective approach for most product manufacturers to explore new sales. However, with the development of a low-carbon economy and the increase in consumers’ low-carbon preference in recent years, some researchers have been interested in questions concerning both dual-channel supply chain and carbon emission issues, especially cap-and-trade regulation [29,30,31,32,33]. Carrillo et al. [29] investigated a dual-channel model that analyzes the impact of consumer environmental sensitivity on a dual-channel supply chain in which the retailer can access both online and retail channels. Zhang et al. [34] analyzed an ecological problem in the field of the dual-channel supply chain. They assumed the manufacturer and retailer sequentially decided to sell low-carbon or standard products in a single or dual-channel model. Nevertheless, the cap-and-trade regulation policy is not mentioned in this research. In terms of cap-and-trade regulation in the dual-channel supply chain, Ji et al. [30] mainly developed dual-channel models to investigate supply chain members’ pricing and carbon emissions reduction decisions under cap-and-trade regulation and retailer promotions. They pointed out that the joint decision on carbon emissions is better in a dual-channel scenario. We take the carbon emission strategy of the dual-channel supply chain into account, and we also consider two different products in the encroachment strategies.

Similarly, Ji et al. [31] investigated an O2O dual-channel with and without cap-and-trade regulation. They also analyzed the impact of consumers’ low-carbon preference on the supply chain member’s decisions. In addition, they proved that the unit carbon quota played a critical role in supply chain members’ decisions. Our paper is relevant to their research, but we consider the impact of the online platform. Xu et al. [32] discussed the impact of low-carbon preference and channel substitution on the decision and coordination in a dual-channel supply chain. Furthermore, Qi et al. [33] considered a dual-channel supply chain coordination under a carbon cap-and-trade regulation. They highlighted the online channel price discount coordination and offline channel price discount contracts to coordinate the dual-channel supply chain. In the same vein, Xu et al. [8] provided an in-depth analysis of the channel addition problem of a manufacturer who has applied cap-and-trade regulation to choose one channel between the marketplace and reselling channel to sell products. Differently, we focus on the impact of consumers’ low-carbon preference and product differentiation between two channels.

As seen from the literature above, the existing research on the dual-channel supply chain management, especially manufacturer’s encroachment scenarios under low-carbon environments, is less reported. Our research contributes to this field in three aspects. First, the above literature seldom analyses the impact of initial unit carbon emission level and carbon quota on the manufacturer encroachment decision. We know the initial carbon emissions and carbon price are both the key factors in the cap-and-trade regulation (Wang et al. [35], Kushwaha et al. [36]), but it is necessary to analyze these two factors together in the manufacturer encroachment issue. Second, we consider the online platform in our model, which is not considered in their work. In real life, online platforms create conditions for encouraging manufacturers to encroach into the market (Chen et al. [12]). Finally, few of the above literature considers two substituted products in the market, which is worth studying. Our model will delve into the optimal decisions in a dual-channel supply chain considering consumers’ low-carbon preferences with and without cap-and-trade regulation. Table 1 summarizes how the proposed paper considerably differs from existing papers.

## 3. Modeling Framework

### 3.1. Supply Chain Structure

**Figure 1:**
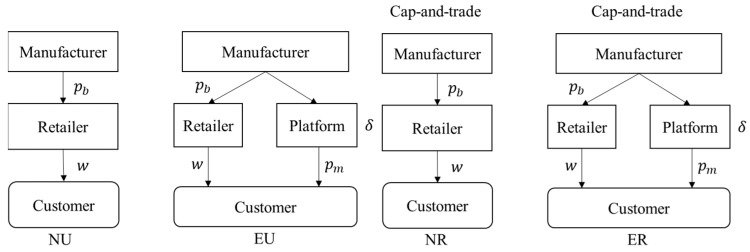
Channel and low-carbon regulation scenarios.

This research investigates a dual-channel supply chain consisting of a manufacturer, a retailer, and an online platform. The retailer delegates the selling of NB products to the consumer by the retailing channel and outsources the producing business to the manufacturer [12]. Considering the practical example, we assume that the manufacturer has the capacity to create FB products for encroaching through an online platform, giving them the option to do so or not. Thus, there are two channel strategies that the manufacturer can choose, no-encroachment and encroaching into the market, as shown in Figure 1. The platform would charge a proportion of the commission rate δ (0<δ<1) based on the sales of FB products. This paper considers the case where the commission rate is exogenous because it follows the industry norm and is widely implemented [36,37].

### 3.2. Cap-and-Trade Regulation

There are two free carbon quota allocation mechanisms: grandfathering and benchmarking. Compared with the grandfathering mechanism, the benchmark management is relatively fair and can be more efficient in promoting facilities’ investments in carbon emission reduction [21]. The carbon quota allowance mechanism in this paper is based on benchmarking, and we assume that there are no carbon trade relations between the manufacturer and the retailer. S represents the government’s free carbon allowance cap for manufacturers, $e_{0}$ represents the initial unit carbon emission of a product. Both NB and FB products are produced by the manufacturer, and these two kinds of products share the same production line. Therefore, we assume that the two substituted products’ initial carbon emission level is similar. For simplicity but without loss of generality, we use e to indicate the unit carbon emission reduction level, which is one of the decision variables for the manufacturer. Thus, the actual unit carbon emission is equal to the initial carbon emission minus the carbon emission reduction level, namely $\left(e_{0}-e\right)$. Further, we assume a linear relationship between total carbon emissions and production output. The total carbon emissions are $E=\left(e_{0}-e\right)\left(D_{b}+D_{m}\right)$. Thus, the carbon trading gap is $\left(E-S\right)p_{e}$, where $p_{e}$ represents the unit carbon price. In addition, we only consider carbon emissions from the production process and ignore other parts. To reduce carbon emissions, the manufacturer must invest in low-carbon technologies. Some researchers usually assume the cost of carbon emission reduction is quadratic or at least convex [1]. Another example is Hartman [38], who studied common air pollution census data from 100 thousand manufacturing companies in 37 industries in the United States. They found that the emission reduction cost is quadratic as well. The convex/quadratic emission reduction cost reflects that the emission reduction in the initial period of carbon emission control is relatively easy. Yet, as the emission reduction amount increases, the reduction of carbon emission in the following period would gradually become difficult. For simplicity and without loss of generality, $c\left(e\right)=\frac{te^{2}}{2}$, and parameter t is understood as the cost–benefit of carbon emission reduction. Combining carbon emissions and carbon emission reduction costs, the total carbon emissions cost under cap-and-trade regulation is $F=\left(E-S\right)p_{e}+c\left(e\right)$.

### 3.3. Demand Function

We assume that the consumer’s valuation v of a unit product is heterogeneous and uniformly distributed in the interval [0, 1], that is v~U [0, 1]. In the model, η represents consumers’ low-carbon preference. If only the NB products exist in the market, the manufacturer chooses no encroachment. Therefore, the utility of consumers buying NB products from the retailing channel is $U_{b}=v-p_{b}+\eta e$. In the interval of $\left[p_{b}-\eta e,1\right]$, consumers choose to buy NB products. Therefore, the consumer demand for purchasing NB products is expressed as follows: $D_{b}=1-p_{b}+\eta e$, where η∈[0,1]. When a manufacturer decides to enter the market, an online platform channel is created. In this scenario, customers are split into two groups: those who prefer to purchase NB products through retail channels and those who prefer to purchase FB products through online platforms. Moreover, consumers make choices between two comparable products depending on the price, brand, and carbon emission reduction level. Customers can value more by purchasing NB products from retail channels than purchasing FB products from online channels; this is because they can immediately get the NB product without waiting, and NB products give them a sense of brand ownership. For this economic phenomenon, Chiang [26] introduced consumer channel acceptance θ (0<θ<1) to define the distinction between the retail channel and the direct internet channel. Consumers value online platform channels as v, specifically when they value the retail channel as θv. Meanwhile, k(0<k<1) indicates consumer brand substitution [12,13], which includes the collection of multiple attributes that consumers perceive NB products compared to FB products, perceived quality, or product packaging, etc. When k=1, both brands become perfect substitutes, while the demand for each product becomes independent when k=0. Therefore, kθv represents the value that consumers obtain from purchasing FB products through the online channel. As a result, $U_{b}=v-p_{b}+\eta e$ and $U_{m}=k\theta v-p_{m}+\eta e$ represents the utility that a consumer who purchases NB products from the retailing channel and FB products from the online platform, respectively. The decision of the channel to choose is determined by comparing the utility of the two channels.

Four cases are discussed as follows: (1) if $U_{b}<0,\;U_{m}<0$, the consumer would not purchase; (2) if $U_{b}>U_{m}\geq 0$, the consumer would buy NB products from the retail channel; (3) if $U_{m}>U_{b}\geq 0$, the consumer would buy FB products from the online platform; (4) if $U_{b}=U_{m}\geq 0$, Consumers buy products from both channels with equal utility. There are four different values, including: $v_{b}=p_{b}-\eta e,\;v_{m}=\frac{p_{m}-\eta e}{k\theta },\;v_{bm}=\frac{p_{b}-p_{m}}{1-k\theta }$. When $v_{b}\geq v_{m},\;v_{bm}\geq v_{b}\geq v_{m}$; when $v_{m}\geq v_{b},\;v_{m}\geq v_{b}\geq v_{bm}$. Summarizing the above cases, the market demand for the two products under the manufacturer’s encroachment can be obtained.

(1) When $v_{b}\geq v_{m}$ and $v_{bm}\leq 1,\;v\in \left[v_{m},v_{bm}\right]$ prefers to buy FB products from the online platform, $v\in \left[v_{bm},1\right]$ prefer to buy NB products from the retailing channel. In this case, the constraint condition $\frac{p_{m}-\left(1-k\theta \right)\eta e}{k\theta }<p_{b}\leq 1+p_{m}-k\theta$ is established, and the demand functions are $D_{m}=v_{bm}-v_{m},\;D_{b}=1-v_{bm}$. (2) When $v_{b}\geq v_{m}$ and $v_{bm}\geq 1$, consumers in $v\in \left[v_{m},1\right]$ prefers to buy FB products from the online channel. In this case, the constraint condition $p_{b}\geq p_{m}+\left(1-k\theta \right)$ is established, and consumers would not buy NB products. In this case, $D_{m}=1-v_{m},\;D_{b}=0$. (3) When $v_{b}\leq v_{m}$, and $v_{bm}\leq 1,\;v\in \left[v_{b},1\right]$ prefer to buy FB products from the online platform, and consumers would not buy FB products. In this case, the constraint condition $p_{b}\leq \frac{p_{m}-\left(1-k\theta \right)\eta e}{k\theta }$ is established, and the demand functions are $D_{m}=0$ and $D_{b}=1-v_{b}$. Above all, dual-channel supply chain demand functions are represented as follows:

$$\left(D_{b},D_{m}\right)=\{\begin{array}{cc} 1-p_{b}+\eta e,\;0 & p_{b}\leq \frac{p_{m}-\left(1-k\theta \right)\eta e}{k\theta }\\ 1-\frac{p_{b}-p_{m}}{1-k\theta },\;\frac{p_{b}-p_{m}}{1-k\theta }-\frac{p_{m}-\eta e}{k\theta } & \frac{p_{m}-\left(1-k\theta \right)\eta e}{k\theta }<p_{b}\leq 1+p_{m}-k\theta \\ 0,\;1-\frac{p_{m}-\eta e}{k\theta } & p_{b}\geq 1+p_{m}-k\theta \end{array}$$

### 3.4. Game Sequence

**Figure 2:**
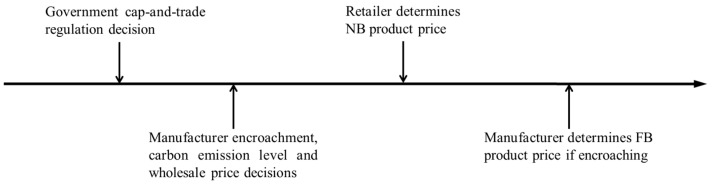
Decision timeline for all members.

The timeline for all chain members’ decisions is depicted in Figure 2. At first, the government decides whether to implement cap-and-trade regulation on the manufacturer. For the no-encroachment scenarios, the following decision sequence is formulated as (1) the manufacturer sets the wholesale price w and the carbon emission reduction level e; (2) the retailer sets the NB products price $p_{b}$; (3) the consumer demand is satisfied by the NB product at the given price.

For the encroachment scenarios, the following decision sequence is formulated as (1) the manufacturer sets the wholesale price w and the carbon emission reduction level e; (2) the retailer sets the NB products price $p_{b}$; (3) the manufacturer sets the price of FB products named $p_{m}$; (4) the consumer demand is satisfied by the NB product at the given price.

This paper studies the optimal decisions of the supply chain in the two scenarios of no encroachment and manufacturer’s encroachment, but we do not consider the situation that only FB products exist in the market, because the manufacturer does not have enough market power to kick its retailer out of the market. In addition, all information in the supply chain is symmetric, and inventory issue is not considered.

## 4. The Main Result

This section derives the equilibrium solutions for the supply chain members in four different models: manufacturer no-encroachment with laissez-faire (NU), manufacturer’s encroachment with laissez-faire (EU), manufacturer no-encroachment with cap-and-trade regulation (NR), and manufacturer’s encroachment with cap-and-trade regulation (ER). Further, we compare the optimal decisions and profits among the four models.

### 4.1. Benchmark Case: No Encroachment with Laissez-Faire (Unregulated Markets) (NU)

We first consider a low-carbon supply chain without cap-and-trade regulation as a benchmark. Based on the demand functions in Section 3, the government has no restrictions on the manufacturer’s carbon emissions, and the manufacturer only chooses to produce NB products. The profit functions of the manufacturer and the retailer are as follows.

$$\pi_{m}=w\left(1-p_{b}+\eta e\right)-\frac{te^{2}}{2};\;\pi_{b}=\left(p_{b}-w\right)\left(1-p_{b}+\eta e\right).\;$$

The manufacturer now decides the wholesale price and carbon emission reduction level by first maximizing its profit. The retailer then determines the optimal NB product price. The backward induction allows us to derive Lemma 1 below.

**Lemma** **1.** *For the Scenario of NU, there exist equilibrium solutions, where* $w^{NU}=w^{NU\#};\;e^{NU}=e^{NU\#};\;p_{b}{}^{NU}=p_{b}{}^{NU\#}$ *.*

We bring the above results into the profit function of NU, and the optimal output and equilibrium profit of supply chain members can be obtained as: $D_{b}{}^{NU}=D_{b}{}^{NU\#}$; $\pi_{m}{}^{NU}=\pi_{m}{}^{NU\#}$; $\pi_{b}{}^{NU}=\pi_{b}{}^{NU\#}$. It can be seen from Lemma 1 that the consumers’ low-carbon preference is one of the essential factors for the manufacturer and the retailer to articulate effective business strategies. When there is no carbon emission regulation in the market, manufacturers would still make some emission reduction measures in case the profits reduction and increase the emission reduction level as consumers’ low-carbon preferences increase. Likewise, the higher the carbon emission reduction level, the higher the wholesale price, the retail price of the NB products, and the profits of supply chain members.

### 4.2. No Encroachment with Regulation (NR)

Under cap-and-trade regulation, the government imposes the cap-and-trade regulation on the manufacturers with a free carbon quota S and unit carbon price $p_{e}$, and the manufacturer chooses to finish producing work for the NB products. In this scenario, the profit functions of the manufacturer and the retailer are shown as follows.

$$\begin{array}{c} {\pi_{m}}^{NR}=w(1-p_{b}+\eta e)-[(e_{o}-e)((1-p_{b}+\eta e))-S]p_{e}-\frac{{te}^{2}}{2},\\ {\pi_{b}}^{NR}=\left(p_{b}-w\right)\left(1-p_{b}+\eta e\right) \end{array}$$

Manufacturers still produce NB products and consider not to encroach into the market. The manufacturer determines the wholesale price and carbon emission reduction level of NB products to maximize their profits according to government regulations. After that, the retailer maximizes profits by deciding on NB product price.

We can get Lemma 2 as follows.

**Lemma** **2.** *For the Scenario of NR with cap-and-trade regulation, there exist equilibrium solutions, where* $w^{NR}=w^{NR\#}$ *,* $e^{NR}=e^{NR\#}$ *,* $p_{b}^{NR}=p_{b}^{NR\#}$ *.*

Bring the above results into the profit function of NR, then the optimal equilibrium results of supply chain members are available: $D_{b}{}^{NR}=D_{b}{}^{NR\#};\;\pi_{m}{}^{NR}=\pi_{m}{}^{NR\#};\;\pi_{b}{}^{NR}=\pi_{b}{}^{NR\#}$. Next, we analyze the sensitivity analyses of consumers’ low-carbon preference impacts on manufacturers’ emission reduction levels, supply chain member’s product pricing strategies, product order volumes, and optimal profits in NR, which are given in Lemma 3.

**Lemma** **3.**
$\frac{\partial e^{NR}}{\partial \eta }>0$ *,* $\frac{\partial w^{NR}}{\partial \eta }>0$ *,* $\frac{\partial D_{b}{}^{NR}}{\partial \eta }>0$ *,* $\frac{\partial \pi_{m}{}^{NR}}{\partial \eta }>0$ *,* $\frac{\partial \pi_{b}{}^{NR}}{\partial \eta }>0$ *.*

Lemma 3 indicates the increase in the manufacturer’s optimal emission reduction level with increasing η. The larger η is, the more sensitive consumers are to the low-carbon products. This means that in the supply chain where the government implements cap-and-trade regulation and the manufacturer does not choose to encroach into the market, consumers’ low carbon preference can effectively encourage the manufacturers to reduce carbon emissions. In addition, the profits of both the manufacturer and the retailer are affected by the low carbon sensitivity coefficient. As consumers become more environmentally conscious, the profits of both the manufacturer and the retailer would increase. However, the cost of producing low-carbon products may increase the wholesale and NB product prices accordingly. As a result, consumers who have a high level of low-carbon preference benefit both the manufacturer and retailer. Therefore, it is an important issue to make more consumers cultivate low-carbon preferences. It is widely believed that government propaganda also has an impact on consumers’ environmental awareness. Therefore, the government could be responsible for raising consumers’ environmental awareness.

### 4.3. Encroachment with Laissez-Faire (EU)

This section discusses supply chain operation strategies when the government decides not to implement cap-and-trade regulations on the manufacturer if the manufacturer chooses to produce NB products for the retailer in addition to FB products through the online platform. When the manufacturer chooses to create FB products, consumers can choose to buy FB brand products or NB products from the retailing channel. Then the profits of the chain members in the dual-channel supply chain are as follows.

$$\pi_{m}=\left(1-\delta \right)p_{m}\left(\frac{p_{b}-p_{m}}{1-k\theta }-\frac{p_{m}-\eta e}{k\theta }\right)+w\left(1-\frac{p_{b}-p_{m}}{1-k\theta }\right)-\frac{te^{2}}{2};\pi_{b}=\left(p_{b}-w\right)\left(1-\frac{p_{b}-p_{m}}{1-k\theta }\right);\;\pi_{p}=\delta p_{m}\left(\frac{p_{b}-p_{m}}{1-k\theta }-\frac{p_{m}-\eta e}{k\theta }\right).$$

**Lemma** **4.** *For the manufacturer encroaching scenario without cap-and-trade regulation, there exists one optimal equilibrium solution, where* $e^{EU}=e^{EU\#}$ *,* $w^{EU}=w^{EU\#}$ *, when* $t>t_{1}$ *.*

Under the equilibrium decisions, the market demand for NB products and the retailer profit are: $D_{b}{}^{EU}=D_{b}{}^{EU\#}$, $\pi_{b}{}^{EU}=\pi_{b}{}^{EU\#}$, respectively. Substituting $e^{EU\#}$, $w^{EU\#}$, $D_{m}{}^{EU\#}$ and $D_{b}{}^{EU\#}$ into $\pi_{b}^{EU}$, $\pi_{m}^{EU}$ and $\pi_{p}^{EU}$, we have $\pi_{b}^{EU}=\pi_{b}^{EU\#}$; $\pi_{m}^{EU}=\pi_{m}^{EU\#}$; $\pi_{p}^{EU}=\pi_{p}^{EU\#}$.

**Lemma** **5.** *(1)* $\frac{\partial e^{EU}}{\partial \eta }>0$ *,* $\frac{\partial w^{EU}}{\partial \eta }>0$ *,* $\frac{\partial D_{b}{}^{EU}}{\partial \eta }<0$ *,* $\frac{\partial D_{m}{}^{EU}}{\partial \eta }>0$ *,* $\frac{\partial \pi_{m}{}^{EU}}{\partial \eta }>0$ *,* $\frac{\partial \pi_{b}{}^{EU}}{\partial \eta }>0$ *,* $\frac{\partial \pi_{p}{}^{EU}}{\partial \eta }>0$ *.*

*(2)* $\frac{\partial e^{EU}}{\partial t}<0$ *,* $\frac{\partial w^{EU}}{\partial t}<0$ *,* $\frac{\partial D_{b}{}^{EU}}{\partial t}<0$ *,* $\frac{\partial D_{m}{}^{EU}}{\partial t}<0$ *,* $\frac{\partial \pi_{m}{}^{EU}}{\partial t}<0$ *,* $\frac{\partial \pi_{b}{}^{EU}}{\partial t}>0$ *,* $\frac{\partial \pi_{p}{}^{EU}}{\partial t}<0$ *.*

Lemma 5 (1) shows that for the scenario where government cap-an-trade regulation does not exist, with the increase in the consumers’ low carbon preference, both the carbon emission level and wholesale price for NB products increase, which also leads to the rise of supply chain members’ profits but the decrease of the quantities of NB products. It states that the whole supply chain members will benefit if consumers are more sensitive to the low-carbon product. In the dual-channel scenario, improving the consumer’s low-carbon awareness is also advantageous for all supply chain members. Lemma 5 (2) highlights that the demand for both NB and FB products, the wholesale price, the manufacturer, and the platform’s optimal profits all decrease with the carbon emission cost coefficient increasing. This finding is intuitive. A higher carbon emission cost leads to a higher encroachment cost and lowers the carbon emission levels for the manufacturer. Thus, the manufacturer will find it challenging to encroach on the market.

### 4.4. Encroachment with Regulation (ER)

Next, we examine the manufacturer encroachment with cap-and-trade regulation. In this scenario, the government imposes cap-and-trade regulations to restrict the carbon emissions of the manufacturer. Meanwhile, the manufacturer chooses to create FB products and encroach into the market through the platform. In this scenario, the profit functions of the manufacturer, the retailer, and the online platform are as follows.

$$\begin{array}{cc} \pi_{m}=\left(1-\delta \right)p_{m} & \left(\frac{p_{b}-p_{m}}{1-k\theta }-\frac{p_{m}-\eta e}{k\theta }\right)+w\left(1-\frac{p_{b}-p_{m}}{1-k\theta }\right)\\ & -\left[\left(e_{o}-e\right)\left(1-\frac{p_{b}-p_{m}}{1-k\theta }+\frac{p_{b}-p_{m}}{1-k\theta }-\frac{p_{m}-\eta e}{k\theta }\right)-S\right]p_{e}-\frac{te^{2}}{2};\;\pi_{b}\\ & =\left(p_{b}-w\right)\left(1-\frac{p_{b}-p_{m}}{1-k\theta }\right);\;\pi_{p}=\delta p_{m}\left(\frac{p_{b}-p_{m}}{1-k\theta }-\frac{p_{m}-\eta e}{k\theta }\right). \end{array}$$

**Lemma** **6.** *For the scenario of manufacturer’s encroachment with cap-and-trade regulation, there exists one optimal equilibrium solution, where* $e^{ER}=e^{ER\#}$ *,* $w^{ER}=w^{ER\#}$ *, when* $t>t_{2}$ *.*

Substituting $e^{ER\#}$, $w^{ER\#}$, $D_{m}{}^{ER\#}$ and $D_{b}{}^{ER\#}$ into $\pi_{b}^{ER}$, $\pi_{m}^{ER}$ and $\pi_{p}^{ER}$, we have $\pi_{b}^{ER}=\pi_{b}^{ER\#}$; $\pi_{m}^{ER}=\pi_{m}^{ER\#}$; $\pi_{p}^{ER}=\pi_{p}^{ER\#}$.

Lemma 6 indicates that the optimal solutions for the manufacturer who are encroaching under cap-and-trade regulations are $e^{ER\#}$ and $w^{ER\#}$. This means that when the government starts to implement cap-and-trade regulation, the optimal carbon emissions $e^{ER\#}$ and wholesale price $w^{ER\#}$ would allow the manufacturer to maximize the profit.

### 4.5. Equilibrium Analysis

In this section, we compare the profits in four models to explore the impact of cap-and-trade regulation and the manufacturer’s encroachment decision.

**Proposition** **1.** *When the government does not implement cap-and-trade regulations, the manufacturer encroaches through the online platform always brings more profits than the no-encroachment scenario, which is* $\pi_{m}^{EU}>\pi_{m}^{NU}$ *. On contract, the retailer loses profits if the manufacturer decides to encroach, which is* $\pi_{b}^{EU}<\pi_{b}^{NU}$ *.*

Proposition 1 demonstrates that encroachment is always a preferred strategy for the manufacturer where the cap-and-trade regulation does not exist. In this traditional way, introducing the FB product into the market has two facts. On the one hand, FB product increases the consumers’ willingness to pay, which allows the manufacturers to extract an enormous surplus and achieve a higher profit via introducing online channels (competition effects). Besides, the retailer’s channel profit shrinks because the manufacturer claims a higher wholesale price for the retailers (wholesale effects). On the other hand, the manufacturer may suffer from the production cost disadvantage and reduced demand for the NB product. Although the manufacturer may face a trade-off between introducing the FB product and production cost disadvantage, without the high carbon emission cost, competition and wholesale effects consistently exceed the production cost increase. Therefore, the manufacturer’s encroachment without cap-and-trade regulation will lead to a win-lose situation.

**Proposition** **2.** *When the government implements cap-and-trade regulation, if the initial carbon emission is within a certain threshold, which is* $e_{0}{}^{\#1}<e_{0}<e_{0}{}^{\#2}$ *, then the manufacturer choosing not to encroach would be the optimal strategy, which is* $\pi_{m}^{ER}>\pi_{m}^{NR}$ *. When the manufacturer’s initial carbon emission is below the threshold* $e_{0}{}^{\#1}$ *or higher than* $e_{0}{}^{\#2}$ *the manufacturer can make more profit by opening an online channel than a single channel, which is* $\pi_{m}^{ER}<\pi_{m}^{NR}$ *.*

Proposition 2 indicates the direct change when the government implements a cap-and-trade strategy, the encroaching decision does not always make the manufacturer better off. It depends on the manufacturer’s initial carbon emission. Compared with a no cap-and-trade regulation scenario, when the initial carbon emission reaches a certain threshold, the profit of no encroachment would be better off. Since lower initial carbon emissions have less impact on the cost of manufacturer’s encroachment, the increase of initial carbon emission, high cost in carbon emission reduction, and the competition from the retailer lead them not to encroach. As initial carbon emissions increase, manufacturers will purchase carbon quotas and encroach into the market again.

**Figure 3:**
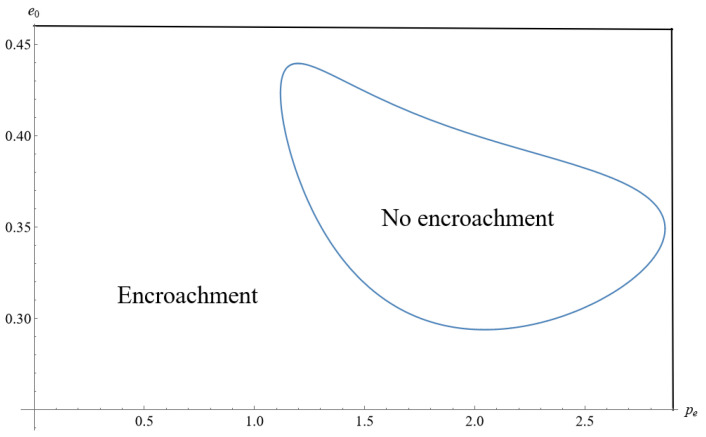
Impact of the initial unit amount of carbon emissions and unit carbon price on manufacturer’s encroachment decision.

To articulate the manufacturer’s encroachment strategy implementing government cap-and-trade regulation, we plot it as a function of $p_{e}$ and $e_{0}$ in Figure 3, assuming that S=0.45, η=0.6, t=3, k=0.75, θ=0.6. Propositions 1 and 2, and Figure 3 confirm that the initial unit amount of carbon emissions and unit carbon price jointly impact the manufacturer’s encroachment decision. Figure 3 demonstrates how the government prevents the factory from encroaching by implementing cap-and-trade regulation when the carbon price is relatively high, and the manufacturer’s initial unit amount of carbon emissions is moderate. For instance, the manufacturer prefers not to encroach because of the high carbon price cost and relatively high or low initial unit amount of carbon emissions. In addition, an increase in the unit carbon price is not always a bad thing for manufacturers who have encroached into the market. Instead, they can improve their emission reduction levels and profit by trading carbon quotas.

**Proposition** **3.** *When the manufacturer decides not to encroach, if* $S\geq S_{1}=\frac{t}{8p_{e}t-2p_{e}\eta^{2}}$ *,* $\pi_{m}^{NR}\geq \pi_{m}^{NU}$ *. Otherwise, there exists the interval of* $e_{0}$ *that if* $e_{0}\in \left(e_{0}{}^{*1},\;e_{0}{}^{*2}\right)$ *,* $\pi_{m}^{NR}<\pi_{m}^{NU}$ *, otherwise,* $\pi_{m}^{NR}>\pi_{m}^{NU}$ *.*

Proposition 3 indicates that when the government carbon quota is sufficient, if the manufacturer chooses not to encroach, implementing the cap-and-trade regulation can make the manufacturer more profitable than not implementing the cap-and-trade regulation. When the government carbon quota is insufficient, if the manufacturer chooses not to encroach, whether the cap-and-trade regulation makes the manufacturer profitable or not depends on the manufacturer’s initial carbon emissions. No cap-and-trade regulation can make the manufacturer more profitable when the manufacturer’s initial carbon emissions are lower than certain thresholds. When a manufacturer’s initial carbon emissions are too low or too high, cap-and-trade regulation can make the manufacturer get more profits. This result is inconsistent with the conclusion of Xue et al. [9]. The author demonstrated that a manufacturer favors the government’s carbon quotas in the single-channel supply chain. However, we show that the manufacturer will benefit from the carbon quotas given by the government not only when the carbon quota is sufficient but also depending on the initial carbon emissions.

**Proposition** **4.** *When the manufacturer decides to encroach, if* $S\geq S_{2}$ *,* $\pi_{m}^{ER}\geq \pi_{m}^{EU}$ *. If* $S<S_{2}$ *, there exists the interval of* $e_{0}$ *that if* $e_{0}\in \left(e_{0}{}^{*3},\;e_{0}{}^{*4}\right)$ *,* $\pi_{m}^{ER}<\pi_{m}^{EU}$ *, otherwise,* $\pi_{m}^{ER}>\pi_{m}^{EU}$ *.*

Proposition 4 indicates that when the government carbon quota is sufficient and the manufacturer chooses to encroach into the market, implementing the cap-and-trade regulation can make the manufacturer more profitable than not implementing the cap-and-trade regulation. When the government carbon quota is insufficient, if the manufacturer chooses not to encroach, whether implementing a cap-and-trade regulation can make the manufacturer profitable depends on the manufacturer’s initial carbon emissions. When the manufacturer’s initial carbon emission is under a threshold, no cap-and-trade regulation can make the manufacturer more profitable. When the manufacturer’s initial carbon emissions are too low or too high, implementing cap-and-trade regulation can make the manufacturer more profitable. It can be easily found that the standard for sufficient carbon quota in the scenario of no-encroachment (Proposition 3) and manufacturer’s encroachment scenario (Proposition 4) is different, that is $S_{1}\neq S_{2}$.

**Proposition** **5.** *(1) When the government does not implement cap-and-trade regulation, manufacturer’s carbon emission reduction levels in the encroachment scenario are higher than no-encroachment scenario, that is* $e^{NU}<e^{EU}$ *always exists.*

*(2) When the government implements cap-and-trade regulation, if the unit carbon price is in the interval of* $p_{e}\in \left[p_{e\left(4\right)},p_{e}{}^{\#}\right]$ *, we can get* $e^{NR}<e^{ER}$ *. Otherwise, we can get* $e^{NR}>e^{ER}$ *.*

Proposition 5 (1) indicates that when the government decides not to implement cap-and-trade regulation, the carbon emission reduction in the dual-channel (encroachment scenario) is always higher than in the single-channel (no-encroachment). Proposition 5 (2) shows that when the government implements cap-and-trade regulation, the manufacturer’s carbon emission reduction level is not associated with the amount of carbon quota, however, it depends on the consumers’ low-carbon preference and the unit carbon price. Specifically, when the consumers’ low-carbon preference is low, the manufacturer’s carbon emission reduction in the non-encroachment scenario is higher. With the consumers’ low carbon preference increasing, manufacturers who choose to encroach would increase the level of carbon emission reduction. In addition, if both the unit carbon price in the government’s cap-and-trade regulation and consumers’ low carbon preference are at a high level, the manufacturer’s carbon emission reduction level in the no-encroachment scenario tends to be higher again.

## 5. Numerical Analyses

This section compares the optimal decisions and profits among the four scenarios to obtain some managerial insights to find which is beneficial to the supply chain members and environment. We focus on the impacts of cap-and-trade regulation, consumers’ low-carbon preference, platform commission rate, and product substitution on two aspects: the firm’s operation decisions and profits. Based on the relevant conditions, this paper selects the value of coefficients widely used in similar literature [31], which can be supposed as follows: t=3,θ=0.6,δ=0.05,k=0.8,S=0.55.

### 5.1. Impact of η on the Carbon Emission Levels and Supply Chain Member’s Profits Considering $e_{0}$

**Figure 4:**
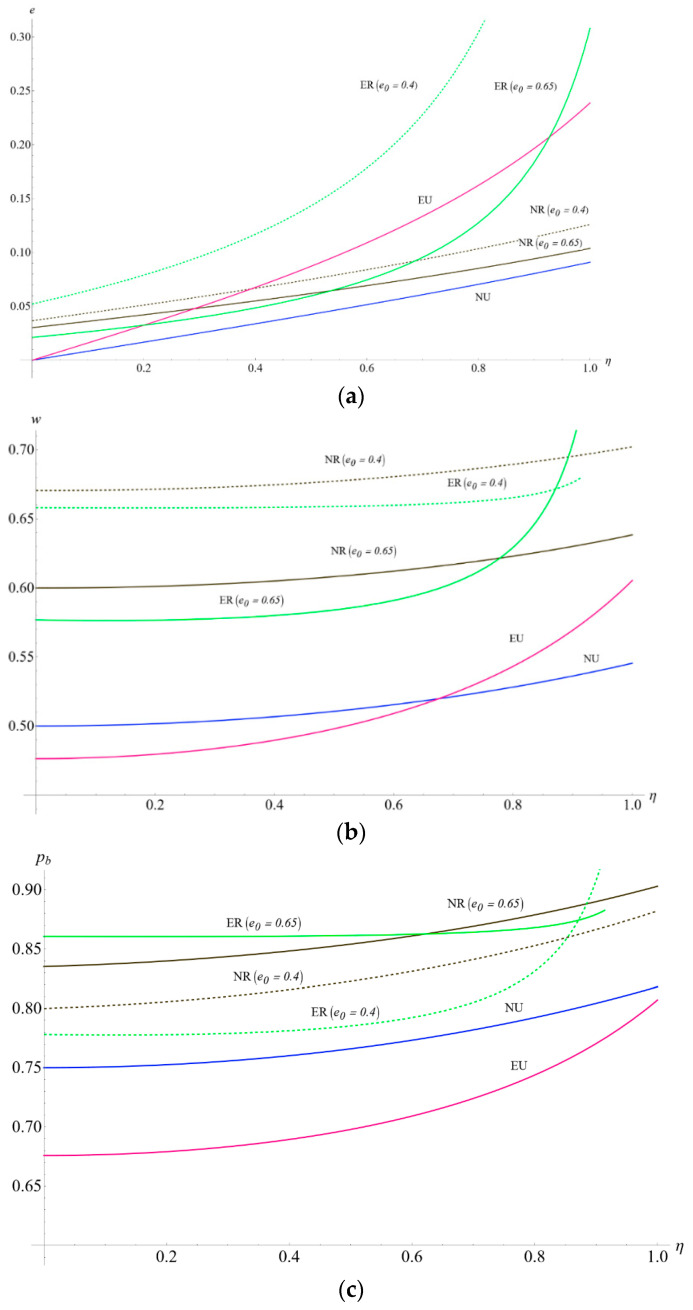

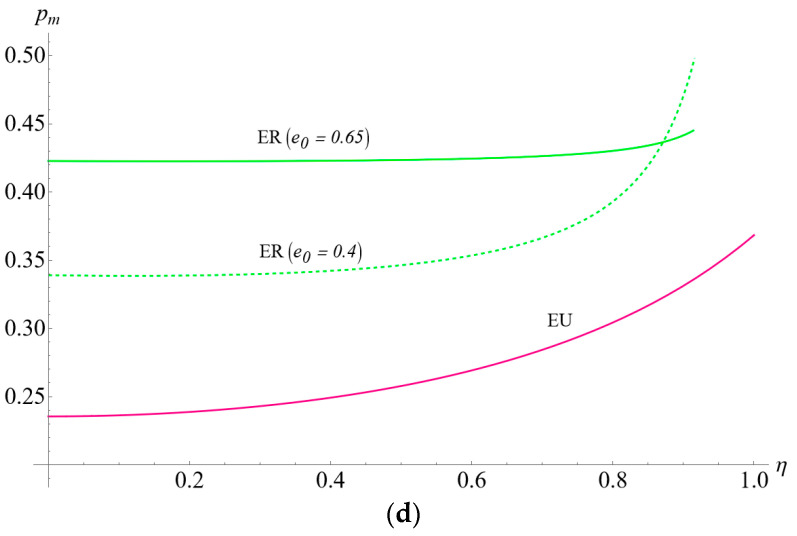
(**a**) The impact of η and $e_{0}$ on the optimal carbon emission levels. (**b**) The impact of η and $e_{0}$ on the optimal wholesale price. (**c**) The impact of η and $e_{0}$ on the NB product price. (**d**) The impact of η and $e_{0}$ on the FB product price.

This subsection presents the impact of consumers’ low-carbon preference and the initial unit amount of carbon emissions from the production process, comparing different initial carbon emission reductions (0.4 versus 0.65). It can be seen from Figure 4a that the increase in consumers’ low-carbon preference witnessed the optimal carbon emission levels increasing in four scenarios, which verify part of the conclusions in Lemmas 3 and 5. Besides, the manufacturer with lower initial carbon emissions is more willing to invest higher costs in improving carbon emission levels. Further, when the initial unit amount of carbon emissions from the production process is relatively low (0.4), the curve of optimal carbon emission level in ER leads to the highest among the four scenarios. When the initial carbon emission is substantial (0.65), the highest carbon emission level in ER is substituted by NR and EU when the consumers’ low-carbon preference is relatively low and moderate, respectively. In addition, with the increase in consumers’ low-carbon preferences, the difference in carbon emission levels between the no-encroachment scenario and encroachment scenario is more evident. Consumers’ low-carbon preference for a dual-channel supply chain could more effectively encourage the manufacturer to reduce carbon emissions than in a single-channel supply chain.

Secondly, Figure 4b–d represents the effect of η and $e_{0}$ on the optimal pricing decisions. Here we initially see that the wholesale price of the supply chain increases with the η in different strategies. This observation verifies Lemmas 3 and 5. Additionally, when the consumer’s low-carbon preference is lower than the threshold value (0.68), $w^{NU}>w^{EU}$. However, this threshold of η increases up to 0.8 with the cap-and-trade regulation. This finding shows that, on the one hand, the government cap-and-trade regulation increases the wholesale price. On the other hand, cap-and-trade regulation increases the threshold of consumers’ low-carbon preference when the wholesale price of the manufacturer’s encroachment is higher than the no-encroachment scenario. This observation indicates that the manufacturer’s encroachment decision can appropriately alleviate the wholesale price pressure with the increasing consumers’ low-carbon preference under the cap-and-trade regulation.

Figure 4c depicts the curves of optimal NB product price in different models concerning η. Like the wholesale price, the retail price will increase with the increasing of consumers’ low-carbon preference. In terms of cap-and-trade regulation, one can easily comment that in the scenarios with cap-and-trade regulation, the NB product price is always higher than the scenarios without cap-and-trade regulation. From the two observations above, it could be concluded that the retailer would continuously improve the NB product price if the government implemented the cap-and-trade regulation in the single retail channel or the dual-channel scenario.

**Figure 5:**
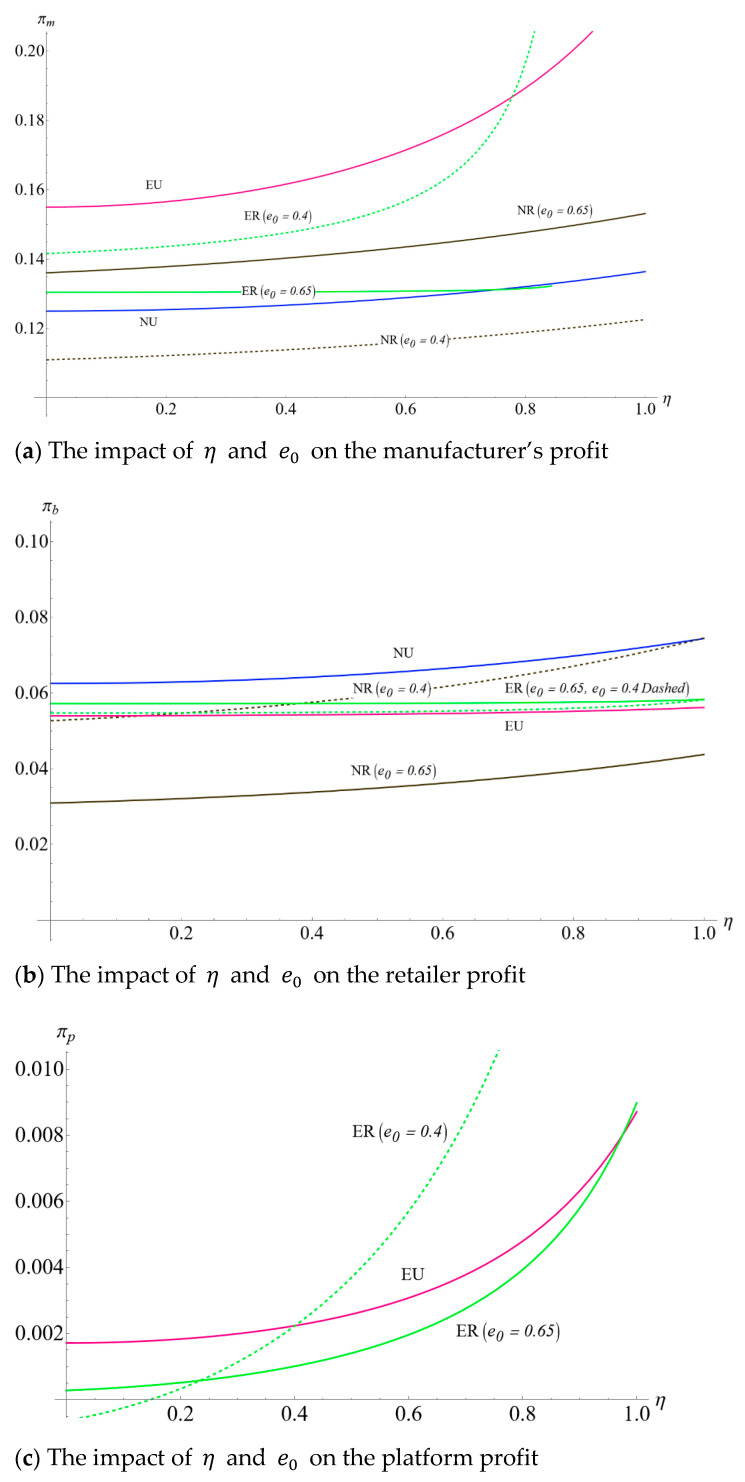
The impact of η and $e_{0}$ on operational decisions and profits.

As Figure 5a shows, the manufacturer’s profit increases with η. When the government implements cap-and-trade regulation, relatively low initial carbon emissions and high consumers’ low-carbon preference will benefit the manufacturer by carbon trading compared with no cap-and-trade regulation scenario. This finding is consistent with Ghosh [39].

Figure 5b depicts the relationship between the retailer’s profit and consumers’ low-carbon preference in the supply chain. The curves of the retailer’s optimal profit as a function of η illustrate that the retailer’s profit in the no-encroachment scenario has increased as consumers’ low-carbon preferences increased. However, in the encroachment scenario, the retailer’s profit has remained stable with the increase in consumers’ low-carbon preference. Besides, manufacturer’s encroachment always squeezes the retailer’s profit when the government does not implement cap-and-trade regulations. However, in the scenario with cap-and-trade regulation, manufacturer’s encroachment is more likely to benefit retailers. This is determined by the manufacturer’s initial carbon emissions and consumers’ low carbon preferences. Specifically, when the manufacturer’s initial carbon emission is relatively high ($e_{0}=0.65$), $\pi_{b}^{ER}>\pi_{b}^{NR}$ is established. When the manufacturer’s initial carbon emissions are relatively low ($e_{0}=0.4$), if consumers have a low preference for low-carbon products, the manufacturer’s encroachment can also increase the retailer’s profit. This observation is novel and interesting. Previous literature indicates that the retailer welcomes a higher consumers’ low-carbon preference and lower initial carbon emissions [36]. However, the observation of Figure 5b contrasts with their findings. Our observation indicates that the government’s low carbon regulation will free-ride the retailer and reduce the manufacturer’s production process emissions. The higher the initial carbon emissions of manufacturers, the more carbon emission during the production process of manufacturers will be regulated by the government. Thus, cap-and-trade regulation might be a win-win strategy for both the manufacturer and the retailer in the supply chain encroachment model.

Figure 5c shows that the profits of the online platform increase with the rise of consumers’ low-carbon preferences. Moreover, the manufacturer who encroaches with lower initial carbon emission is more beneficial for the platform and vice versa. It indicates that the platform should enhance the environmental supervision of its suppliers. For example, JD.com has established environmental inspection standards for its suppliers, including the “Green Procurement Management Regulations.” In this regulation, suppliers must mention their environmental protection capabilities and contributions. In 2020, 85% of new suppliers were screened according to these environmental standards [40].

### 5.2. Impact of η and $p_{e}$ on the Carbon Emission Levels

**Figure 6:**
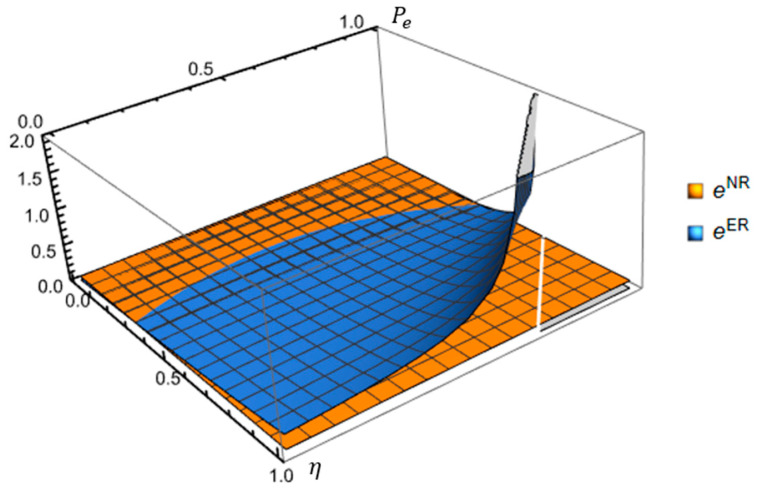
Impact of η and $p_{e}$ on the carbon emission levels.

From Figure 6, we observe that the consumer’s low-carbon preferences and carbon price jointly impact manufacturers’ carbon emission reduction levels. In the no-encroachment scenario, the carbon reduction level increases slowly. In the encroachment scenario, with the consumers’ low-carbon preference and carbon price increasing, carbon emission reduction levels keep steady and then increase rapidly. The manufacturer prefers increasing carbon emission reduction levels. Besides, when the consumers’ low-carbon preference is low, $e^{ER}<e^{NR}$ always exists. When the carbon price and carbon emission reduction are higher than some thresholds, the excessive unit carbon price will cause manufacturers to drop out their investment in carbon emission reduction. This is because manufacturer’s encroachment will increase their cost in increasing carbon emission reduction levels and make them unprofitable. Instead, manufacturers will choose to purchase a carbon quota. Figure 6 has verified part of Proposition 5.

### 5.3. The Impact of $e_{0}$ and $p_{e}$ on the Optimal Manufacturer’s Profits When the Total Carbon Quota Is Insufficient (S=0.15) and Sufficient (S=0.45)

**Figure 7:**
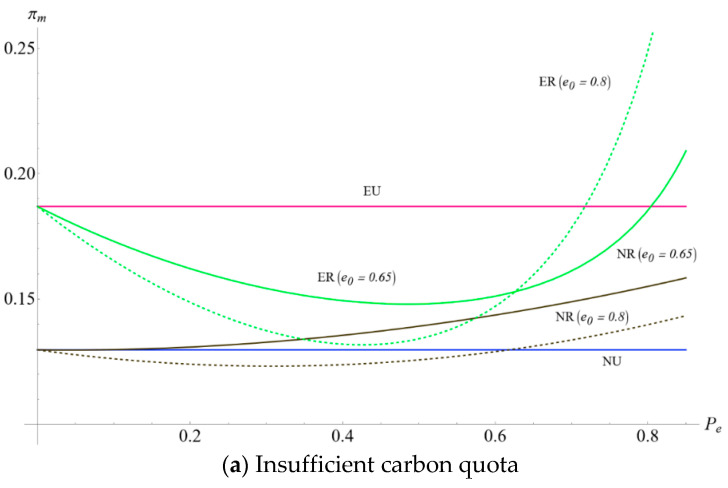

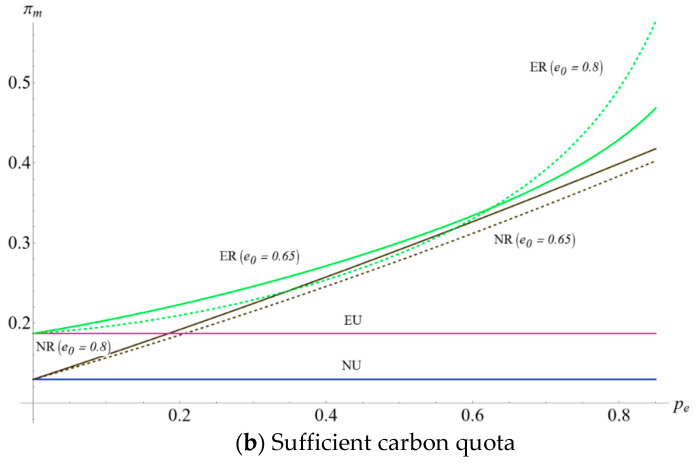
The impact of $e_{0}$ and $p_{e}$ on the optimal manufacturer’s profits when the total carbon quota is insufficient (S=0.15 ) and sufficient (S=0.45 ).

It can be seen from Figure 7a,b that Scenario ER always dominates scenario NR, and Scenario EU always dominates Scenario NU in terms of the manufacturer’s optimal profits. It indicates that the encroachment strategy continuously improves manufacturers’ optimal profits whether the government implements cap-and-trade regulation or not. This conclusion verifies part of Proposition 1 and Proposition 2. Moreover, it is intriguing to note from Figure 7a that the increase of the unit carbon price witnesses the manufacturer’s optimal profit first decrease and then increase in Scenario ER with insufficient total carbon quota. In other words, there is a trade-off between investing in carbon emissions and purchasing a carbon quota if the carbon quota from the government is insufficient. When the $p_{e}$ is relatively low, the manufacturer manages to purchase the carbon quota rather than improving the carbon emission level. When the unit carbon price is much higher, the manufacturer is more willing to invest in producing low carbon production. When the carbon quota is sufficient, from Figure 7b, we can see that the manufacturer’s optimal profits in Scenario ER and Scenario NR increase with $p_{e}$. The manufacturer can incur additional profits in this scenario by trading excess carbon [41]. Additionally, combing Figure 7a,b, we observe a counterintuitive but interesting result that cap-and-trade regulation may not bring more profit than the laissez-faire market for the manufacturer. This phenomenon appears in both single-channel and dual-channel scenarios. The insufficient carbon quota and relatively high initial unit amount of carbon emissions from the production process would incur a high cost for the manufacturer. Cap-and-trade regulation with a sufficient carbon quota is always beneficial for the manufacturer. In contrast, an insufficient carbon quota reduces the manufacturer’s profit at first but increases at the end. In practice, some governments (such as those in China and the European Union) gradually increase the unit carbon price in the carbon trading markets. This policy seems to be hard at first and then easily implemented. This finding verifies the conclusion in Proposition 4.

### 5.4. Impact of Cost Coefficient of Emission Reduction in Different Scenarios

**Figure 8:**
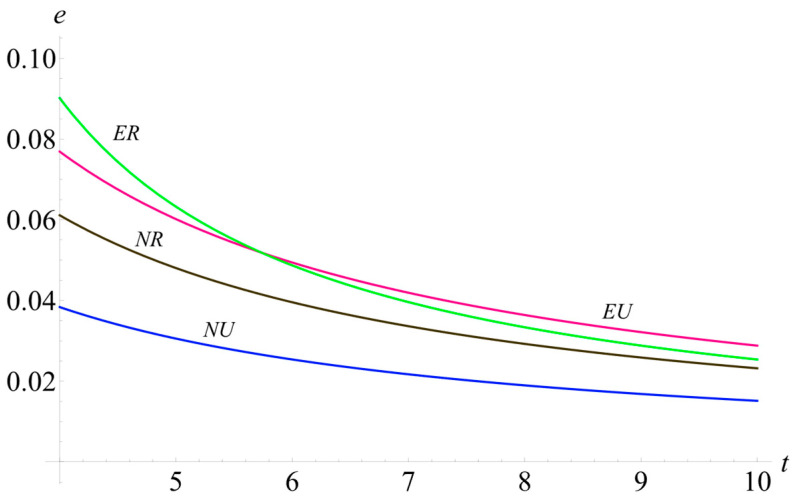
The impact of the cost coefficient of emission reduction on the carbon emission reduction levels.

Figure 8 depicts the impact of the cost coefficient of emission reduction on the carbon emission reduction levels. It can be seen from Figure 8 that the carbon emission level in four scenarios decreases in the cost coefficient of emission reduction. More specifically, the NU is the lowest, and the ER is higher than NR. However, the gap between ER and NR is getting close with the increasing cost coefficient of emission reduction. ER and EU are always higher than NR and NU. It indicates that encroachment may require the manufacturer to improve higher carbon emission reduction levels compared with no encroachment scenarios. However, the increasing cost coefficient of emission reduction would reduce the manufacturer’s investment in carbon emission level improvement. Instead, they tend to purchase more carbon quotas.

### 5.5. Impact of Consumer’s Online Channel Preference in Different Scenarios

**Figure 9:**
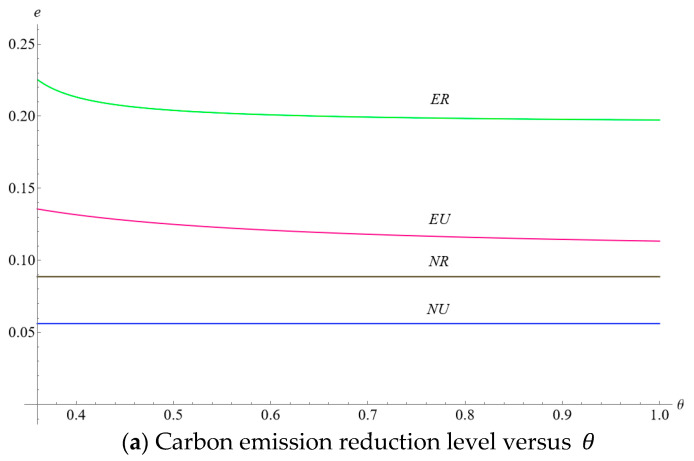

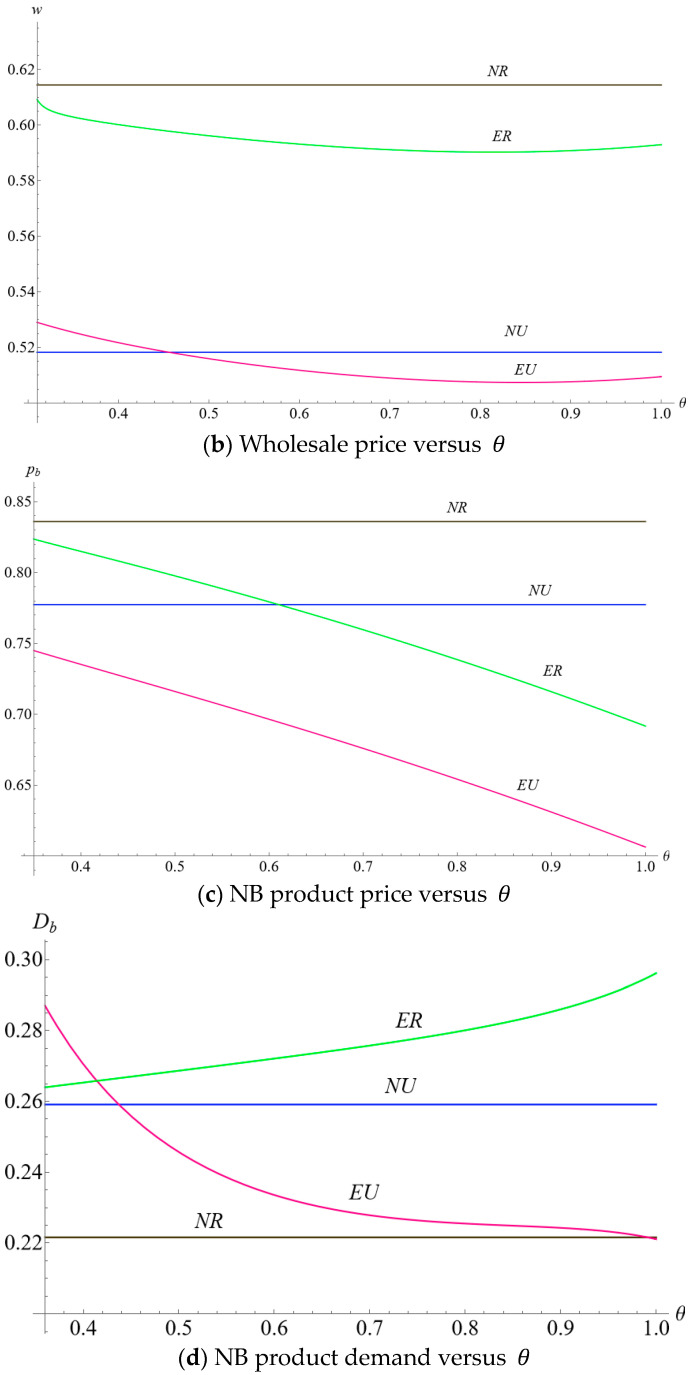

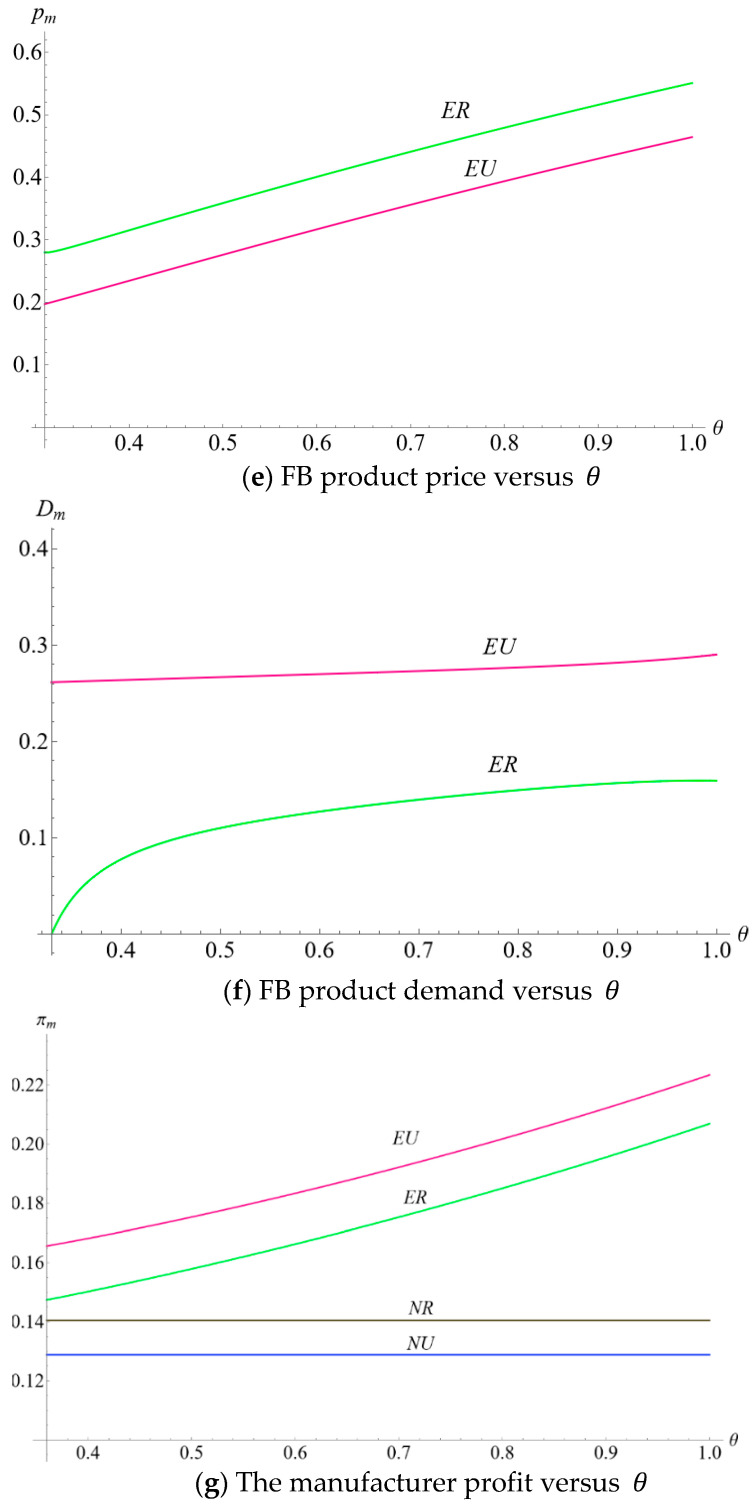

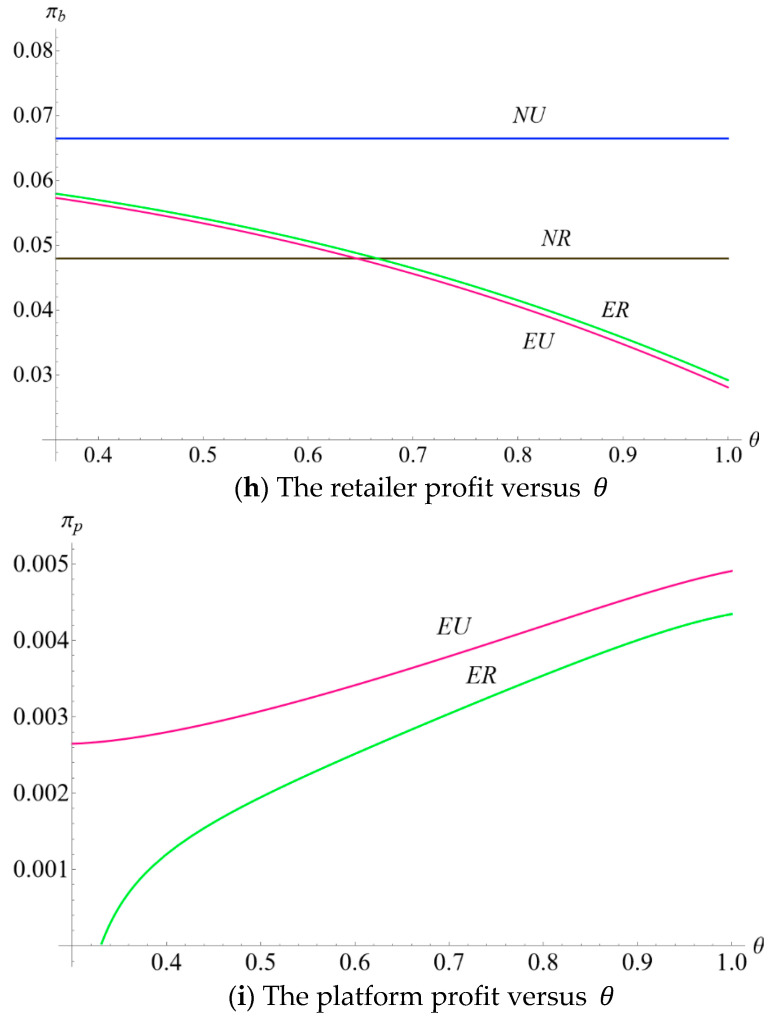
Optimal equilibrium versus θ.

We now analyze the impact of consumers’ online channel preferences on different scenarios. Given the parameters’ setting, Figure 9a–i illustrate the comparison results of equilibrium outcomes of four scenarios. From Figure 9a–i, we can get several insights as follows.

In Figure 9a, compared with laissez-faire scenarios, cap-and-trade regulation raises the firm’s carbon emission reduction level. Accordingly, opening an online channel always requires the manufacturer to invest more cost into carbon emission reduction activity. Figure 9b shows the wholesale price versus consumer online channel preference. As evidenced in Figure 9b, when θ increases, the optimal wholesale price in ER and EU decreases. Besides, a threshold value of parameter θ determines the relation of the wholesale price between two single-channel scenarios. If the parameter θ is sufficiently small such that the parameter is smaller than 0.46, the wholesale price of scenario NU keeps stable and lower than scenario EU. However, with the increase in the consumer online channel preference, the wholesale price of the EU reduces and then is lower than EU. With the increasing parameter θ, reducing the wholesale price could help the manufacturer to get the optimal profits, as well as reducing the channel conflict with the retailers.

Figure 9c,d shows the trend of NB product demand and price change. In terms of the NB products demand, in the scenario ER, with the increasing of consumer online channel preference, NB product demand is increasing rather than decreasing. Meanwhile, in the Scenario EU as the contract, we observe that NB’s demand reduces dramatically. This result is counterintuitive but interesting. Normally, the more consumer prefers online channel, the less demand would retailer channel have. However, in the scenario with government cap-and-trade regulation, the NB product increases their demand. Accordingly, Figure 9h shows the profit change of the retailer, in which the retailer could benefit from the government implementing cap-and-trade regulation. In addition, some threshold values for parameter θ determine the relations of NB product price (as well as NB product demand), wholesale price, and profits. For example, in terms of NB product demand (Figure 9h), if the online channel preference is relatively low, the ER and EU are higher than NR, which means that manufacturer encroachment decision is not always harmful to the retailer. Above all, the retailer may welcome their supplier to add a new channel and compete with them, especially when the government implements cap-and-trade regulation to some extent.

Figure 9e–g illuminates the effects of the parameter θ on the manufacturer’s optimal equilibrium, including FB product price, demand, and the manufacturer’s profit. The results show that by increasing parameter θ, FB product price of EU and ER increases, and the price of ER is higher than EU. FB product demand for scenario ER increases as well, but FB product demand for Scenario EU is almost fixed and is not sensitive to parameter θ. Moreover, according to Figure 9g, the manufacturer’s profit of scenario ER and EU slightly increase with consumer online channel preference. Higher online channel preference would attract more consumers to switch from retail to online platform channel. Figure 9h illustrates the impact of consumer online channel preferences on retailer profits. Figure 9h shows two perspectives. On the one hand, the manufacturer’s encroachment always hurts retailer profits without cap-and-trade regulation. On the other hand, when the consumer online channel preference is lower than a specific threshold value (approximate 0.68), encroachment may benefit the retailer. Therefore, retailer managers should pay attention to the attractiveness of their retailing channels to prevent loss of demand and profits in the low-carbon environment. Finally, Figure 9i demonstrates that the platform profit increases as more consumers prefer the online channel. This result is intuitive. As the consumer preference for online platforms increases, online consumer demand would consequently increase.

### 5.6. Impact of Product Substitutions and Commission Rates in the Two Encroachment Scenarios

Data from Table 2 shows the equilibrium outcomes of the encroachment scenario, which numerically demonstrate the two different environmental regulation models (with and without cap-and-trade regulation). Here, k=0.4 and k=0.7 represent the situation where FB products have relatively low and similar consumer brand preferences compared to NB products. θ=0.5 and θ=1 are taken by considering that consumers have a relatively low and equal online channel preference compared to the retailer channel. Meanwhile, the supply chain’s optimal equilibrium in different platform commission rates is also tabulated. We have checked all the data which are necessary for the existence of the optimal equilibrium. Observations are listed as follows.

From Table 2, we note that a higher online channel preference increases the market demand with cap-and-trade regulation. However, it decreases the NB product demand without cap-and-trade regulation. In addition, increasing the online channel preference leads to both the manufacturer and the platform profit increase, but the retailer profit decreases. This observation holds with/without cap-and-trade regulation and verifies the conclusion in the 5.4 Subsection. Besides, comparing Table 2a,b, cap-and-trade regulation increases carbon emissions reduction levels, profits of the manufacturer, the retailer, and the online platform. However, the platform’s profits would slightly decrease when both products become more substitutes. Further, with consumer brand substitution increases, the wholesale price will decrease. Meanwhile, the pricing of two products and the manufacturer’s profit growth. It is worth noting that online platform managers should be cautious in dealing with manufacturer’s encroachment if the FB products strongly resemble the NB products. This finding may be close to part of the conclusion of Chen [12]. We have also verified that this conclusion is right in a low-carbon environment. In terms of commission rate, the rise of the platform commission may make the platform profit increase first and then decrease. The manufacturer’s profit will also be affected by the rise of the platform commission rate. On the other hand, retailer profits will increase due to the rising commission rate. Therefore, an excessively higher commission rate may not benefit the online platform because it would increase the cost of manufacturer’s encroachment and reduce the manufacturer’s demand.

## 6. Discussion

From Table 1, we can see that most of the existing research related to this paper considers the dual-channel supply chain considering cap-and-trade regulation. However, most existing research does not consider the online platform and only one product in the market. Our research explores the online platform and two different products in the manufacturer encroachment model to make our research more realistic. Yang et al. [22] jointly studied the manufacturer’s optimal channel selection and carbon emission reduction decision with and without cap-and-trade regulation. Our study defines how the consumers’ low-carbon preference impacts the manufacturer’s carbon emission reduction level, supply chain members’ operation decisions, and equilibrium profits.

The authors of the article (Xu et al. [8], Zhang et al. [34]) in Section 2 claim that encroaching decisions with cap-and-trade regulation may always make the manufacturer better off. Nevertheless, the authors of the article (Ji et al. [24], Xu et al. [18], Yang et al. [22]) believe that adding a new selling channel may not always benefit the manufacturer. Our research verified that when the government implements a cap-and-trade strategy, the encroaching decision does not always make the manufacturer better off. It depends on the carbon quota, the manufacturer’s initial carbon emission and the unit carbon price. Our research also obtains the relevant threshold value of these three factors. Therefore, the conclusion of this paper may have a breakthrough based on all the above articles. In terms of the carbon emission reduction result, the author of the article (Xue et al. [9], Xu et al. [32], Ji et al. [30]) believes that increasing consumers’ low-carbon preference can always help the manufacturer reduce carbon emissions. This paper determines the threshold of the carbon price and consumers’ low-carbon price and compares the carbon emission reduction level in the encroachment and no-encroachment scenarios.

Further, the authors of the article (Yu et al. [25], Xu et al. [8], Xu et al. [18]) think that increasing the platform commission rate will not increase the platform profit. This paper investigates that the rising platform commission rate may cause the platform profit to increase first and then decrease. In this paper, the above three different research conclusions are combined and improved, researching cap-and-trade regulation and online platforms.

## 7. Conclusions

This paper develops models to investigate the manufacturer’s encroachment and carbon emission reduction decisions with or without government cap-and-trade regulation. The manufacturer not only produces national brand products for the retailer, but also has a chance to sell factory brand products through the online platform. Depending on whether the government implements cap-and-trade regulation or not, four scenarios for different supply chain structures are considered, namely, no-encroachment laissez-faire (NU), no-encroachment with cap-and-trade (NR), encroachment laissez-faire (EU), and encroachment with cap-and-trade (ER). We have the following findings from the analytical comparison and numerical study:

(1) The encroachment decision is always profitable for the manufacturer when the government decides not to implement cap-and-trade regulation, and the retailer always loses profit. When the carbon quota is sufficient, cap-and-trade regulation is always beneficial to the manufacturer because trading excess carbon quota can gain more profit. Moreover, when the government’s carbon quota is insufficient, the manufacturer’s encroachment decision depends on the initial unit amount of carbon emissions and unit carbon price. As a result, an increase in the unit carbon price is not always bad for the manufacturer who has encroached into the market. Managers of the manufacturer should care about the initial unit amount of carbon emissions and the unit carbon price policy to avoid profit decrease.
(2) Consumers’ low-carbon preference and the unit carbon price have a joint impact on carbon emission reduction levels. The carbon emission reduction in encroachment is always higher than in the no-encroachment scenario without cap-and-trade regulation. The manufacturer’s carbon emission reduction in the no-encroachment scenario is higher than in the encroachment scenario if the consumers’ low-carbon preference is low. With the consumers’ low-carbon preference increasing, manufacturers who encroach would have higher carbon emission reduction. Unless the unit carbon price exceeds a certain threshold, relatively high consumer’s low-carbon preference in the encroachment supply chain could more effectively encourage the manufacturer to reduce carbon emissions than in the no-encroachment supply chain.
(3) Consumers with a high level of low-carbon preference can benefit all three members. Additionally, under the cap-and-trade regulation, the manufacturer’s encroachment decision can alleviate the wholesale price pressure with the increasing consumers’ low-carbon preference. This conclusion indicates that making more consumers cultivate low-carbon consciousness is an important issue because it benefits both the chain members’ profit and the carbon neutrality achievement of countries. The online platform’s profit increase with the consumer’s low-carbon preference. It indicates that online platform managers should enhance the environmental supervision of their suppliers.
(4) The commission rate, online channel preference, and product substitution influence the platform’s profits. For commission rates, platform profit to increase first and then decrease if the commission rate is relatively high. For channel preference, higher online channel preference would attract more consumers to switch from retail to online platforms, and the platform profit increases with more consumers prefer the online channel. For product substitution, cap-and-trade regulation increases the chain members’ carbon emissions reduction levels and profits. However, it would slightly decrease platform profits if both FB and NB products become more substitutes.

We offer several comparisons of four different scenarios in terms of chain members. (see Table 3).

Although this research is well-sustained by the literature and integrates manufacturer’s encroachment decision and government cap-and-trade regulations, there are still some limitations due to the assumptions that can be relaxed in future research. In this paper, we have assumed only the manufacturer lead Stackelberg game model. One can extend this work by assuming a retailer lead game model. Additionally, the manufacturer may not observe the FB product’s demand before making encroachment decisions. Thus, integrating the demand uncertainty into the model would be worth studying.

## Figures and Tables

**Table 1:** Comparison between the existing models in the relevant literature and this research.

Papers	Dual Channel	Platform	Cap-and-Trade Regulation	Products
Anand et al., 2020 [1]	No	No	Yes	n
Ji et al., 2017 [31]	Yes	No	Yes	1
Chen et al., 2019 [12]	Yes	No	No	2
Liu et al., 2020 [23]	No	Yes	Yes	1
Xu et al., 2022 [18]	No	Yes	Yes	1
Xu et al., 2021 [8]	Yes	Yes	Yes	1
Xu et al., 2018 [32]	Yes	No	Yes	1
Yang et al., 2018 [22]	Yes	No	Yes	1
Yu et al., 2022 [25]	No	Yes	Yes	1
Drake et al., 2016 [5]	No	No	Yes	n
Zhang et al., 2020 [34]	Yes	No	No	2
This research	Yes	Yes	Yes	2

**Table 2:** Equilibrium outcomes of the encroachment scenario.

k	θ	δ	$e^{EU}$	$w^{EU}$	$p_{b}{}^{EU}$	$p_{m}{}^{EU}$	$Q_{b}{}^{EU}$	$Q_{m}{}^{EU}$	$\pi_{r}{}^{EU}$	$\pi_{m}{}^{EU}$	$\pi_{p}{}^{EU}$
(a) Encroachment occurs without cap-and-trade regulation											
0.4	0.5	0.05	0.134	0.526	0.716	0.163	0.328	0.259	0.06	0.16	0.0027
		0.1	0.126	0.521	0.717	0.164	0.299	0.262	0.061	0.156	0.0049
		0.2	0.111	0.511	0.721	0.166	0.237	0.269	0.064	0.15	0.0079
		0.3	0.098	0.501	0.748	0.17	0.166	0.277	0.068	0.145	0.0085
	1	0.05	0.112	0.509	0.676	0.273	0.228	0.267	0.053	0.175	0.003
		0.1	0.107	0.503	0.677	0.277	0.205	0.274	0.056	0.171	0.0056
		0.2	0.096	0.49	0.682	0.286	0.151	0.29	0.062	0.162	0.0085
		0.3	0.086	0.478	0.709	0.294	0.086	0.308	0.071	0.154	0.0076
0.7	0.5	0.05	0.114	0.512	0.687	0.243	0.238	0.265	0.055	0.189	0.0029
		0.1	0.109	0.506	0.688	0.246	0.214	0.271	0.058	0.171	0.0053
		0.2	0.09	0.494	0.693	0.253	0.161	0.284	0.063	0.159	0.0081
		0.3	0.088	0.483	0.719	0.264	0.097	0.299	0.071	0.151	0.0036
	1	0.05	0.104	0.502	0.6	0.415	0.216	0.351	0.036	0.21	0.0045
		0.1	0.1	0.492	0.602	0.421	0.183	0.349	0.042	0.202	0.0077
		0.2	0.09	0.474	0.609	0.437	0.107	0.345	0.056	0.189	0.0093
		0.3	0.082	0.457	0.641	0.46	0.015	0.398	0.073	0.177	0.002
(b) Encroachment occurs with cap-and-trade regulation											
0.4	0.5	0.05	0.293	0.602	0.834	0.242	0.26	0.41	0.06	0.275	0.005
		0.1	0.404	0.599	0.834	0.246	0.265	0.717	0.062	0.267	0.018
		0.2	0.619	0.58	0.823	0.243	0.274	1.367	0.067	0.24	0.067
		0.3	0.788	0.548	0.799	0.225	0.282	1.954	0.071	0.202	0.132
	1	0.05	0.175	0.591	0.792	0.353	0.268	0.11	0.05	0.295	0.002
		0.1	0.157	0.587	0.796	0.362	0.278	0.051	0.058	0.292	0.002
		0.2	0.118	0.583	0.806	0.386	0.299	0	0.067	0.286	0.006
		0.3	0.073	0.583	0.827	0.422	0.325	0	0.079	0.284	0.034
0.7	0.5	0.05	0.17	0.593	0.803	0.326	0.266	0.559	0.056	0.291	0.002
		0.1	0.147	0.59	0.806	0.335	0.022	0.274	0.06	0.287	0.001
		0.2	0.098	0.587	0.818	0.358	0	0.29	0	0.283	−0.01
		0.3	0.04	0.59	0.839	0.394	0	0.315	0	0.285	−0.04
	1	0.05	0.182	0.585	0.72	0.502	0.284	0.155	0.037	0.33	0.004
		0.1	0.172	0.578	0.721	0.513	0.309	0.105	0.044	0.323	0.005
		0.2	0.151	0.566	0.734	0.544	0.365	0	0.06	0.31	0.001
		0.3	0.127	0.559	0.758	0.588	0.433	0	0.087	0.302	0.029

**Table 3:** Differences between the four scenarios in terms of chain members.

Four Different Scenarios	Details
NU	The manufacturer will not choose this strategy comparing with EU. Retailer welcomes this strategy comparing with EU.
NR	The manufacturer will choose depending on carbon quota. Retailer welcomes this strategy comparing with ER.
EU	The manufacturer always gets profits. The manufacturer reduces more carbon emission comparing with NU. Online platform welcomes this strategy.
ER	The manufacturer achieves profits if carbon quota is sufficient, or it depends on the initial unit amount of carbon emissions and unit carbon price. The manufacturer reduces more carbon emission if consumers’ low-carbon preference is high compared with NR. Online platform should enhance the environmental supervision of their suppliers and implement a moderate commission rate.

## Data Availability

Not applicable.

## Supplementary Materials

The following supporting information can be downloaded at: https://www.mdpi.com/article/10.3390/ijerph191610407/s1.
