# Supplementary material for: Manufacturer’s Encroachment and Carbon Emission Reduction Decisions Considering Cap-and-Trade Regulation and Consumers’ Low-Carbon Preference
Source: Int J Environ Res Public Health. 2022 Aug 21;19(16):10407. doi: 10.3390/ijerph191610407 (PMC9408328; doi:10.3390/ijerph191610407)
Supplement: Supplementary file 1 [file ijerph-19-10407-s001.zip › applendix.pdf]

Appendix

Equilibrium result

$$w^{NU\#}=\frac{2t}{4t-\eta^2},\ e^{NU\#}=\frac{\eta}{4t-\eta^2},\ {p_b}^{NU\#}=\frac{3t}{4t-\eta^2},\ {D_b}^{NU\#}=\frac{t}{4t-\eta^2},\ {\pi_m}^{NU\#}=\frac{t}{8t-2\eta^2},\ {\pi_b}^{NU\#}=\frac{t^2}{(4t-\eta^2)^2}.$$

$$w^{NR\#}=\frac{2t-p_e\left(p_e+\eta+e_op_e\eta-e_o(2t-\eta^2)\right)}{4t-(p_e+\eta)^2}\ ,\quad e^{NR\#}=\frac{(1-e_op_e)(p_e+\eta)}{4t-(p_e+\eta)^2}\ ,\quad p_b^{NR\#}=\frac{3t-p_e\left(p_e+\eta+e_op_e\eta-e_o(t-\eta^2)\right)}{4t-(p_e+\eta)^2}\ ,$$

$${D_b}^{NR\#}=\frac{(1-e_op_e)t}{4t-(p_e+\eta)^2},\ {\pi_m}^{NR\#}=\frac{(1-e_op_e)^2t}{2(4t-(p_e+\eta)^2)}+p_eS,\ {\pi_b}^{NR\#}=\frac{(1-e_op_e)^2t^2}{(4t-(p_e+\eta)^2)^2}.$$

$$e^{EU\#}=$$

$$\frac{k(-1+\delta)\eta\theta(-1+k\theta)(\delta(-6+k\theta)(-2+k\theta)-2(8+k\theta(-5+k\theta)))}{kt\theta(4k(13-\delta)(1-\delta)\theta-32(1-\delta)-4k^2(6-\delta)(1-\delta)\theta^2+k^3(2-\delta)^2\theta^3)-2(1-\delta)\eta^2(1-k\theta)(k\theta(5-k\theta)-8+\delta(4-k\theta)(2-k\theta))}\ ,$$

$$w^{EU\#}=$$

$$\frac{(-1+\delta)(-1+k\theta)((-1+\delta)\eta^2(-4+k\theta)(-2+k\theta)(-1+k\theta)+2kt\theta(-8+k\theta(2(3+\delta)-k(2+\delta)\theta)))}{kt\theta(4k(13-\delta)(1-\delta)\theta-32(1-\delta)-4k^2(6-\delta)(1-\delta)\theta^2+k^3(2-\delta)^2\theta^3)-2(1-\delta)\eta^2(1-k\theta)(k\theta(5-k\theta)-8+\delta(4-k\theta)(2-k\theta))},$$

$${D_m}^{EU\#}=$$

$$\frac{(2-k\theta)(2-\delta(2-k\theta))(kt\theta(2-k(2-\delta)\theta)-(1-\delta)\eta^2(1-k\theta))}{kt\theta(32(1-\delta)-4k(13-\delta)(1-\delta)\theta+4k^2(6-\delta)(1-\delta)\theta^2-k^3(2-\delta)^2\theta^3)-2(1-\delta)\eta^2(1-k\theta)(8-k\theta(5-k\theta)-\delta(4-k\theta)(2-k\theta))},$$

$${D_b}^{EU\#}=$$

$$\frac{(1-\delta)\eta^2(3-k\theta)(2-k\theta)(1-k\theta)+2kt\theta(2-2k\theta-\delta(2-k\theta)(3-2k\theta))}{kt\theta(32(1-\delta)-4k(13-\delta)(1-\delta)\theta+4k^2(6-\delta)(1-\delta)\theta^2-k^3(2-\delta)^2\theta^3)-2(1-\delta)\eta^2(1-k\theta)(8-k\theta(5-k\theta)-\delta(4-k\theta)(2-k\theta))},$$

$$\pi_b^{EU\#}=$$

$$\frac{2(2-k\theta)(1-k\theta)(2-\delta(2-k\theta))^2((1-\delta)\eta^2(1-k\theta)-kt\theta(2-k(2-\delta)\theta))^2}{(kt\theta(32(1-\delta)-4k(13-\delta)(1-\delta)\theta+4k^2(6-\delta)(1-\delta)\theta^2-k^3(2-\delta)^2\theta^3)+2(1-\delta)\eta^2(1-k\theta)(-8+k\theta(5-k\theta)+\delta(4-k\theta)(2-k\theta)))^2},$$

$$\pi_m^{EU\#}=$$

$$\frac{(1-\delta)(1-k\theta)((1-\delta)\eta^2(2-k\theta)^2(1-k\theta)-4kt\theta(2-k\delta\theta(2-k\theta)))}{4(1-\delta)\eta^2(1-k\theta)(8-k\theta(5-k\theta)-\delta(4-k\theta)(2-k\theta))-2kt\theta(32(1-\delta)-4k(13-\delta)(1-\delta)\theta+4k^2(6-\delta)(1-\delta)\theta^2-k^3(2-\delta)^2\theta^3)},$$

$$\pi_p^{EU\#}=$$

$$\frac{k\delta\theta(1-k\theta)((1-\delta)\eta^2(2-k\theta)(1-k\theta)+2kt\theta(6\delta+k(8-5\delta)\theta-k^2(2-\delta)\theta^2-10))(2kt\theta(2k\theta-2+\delta(2-k\theta)(3-2k\theta))-(1-\delta)\eta^2(3-k\theta)(2-k\theta)(1-k\theta))}{(2(1-\delta)\eta^2(1-k\theta)(8-k\theta(5-k\theta)-\delta(4-k\theta)(2-k\theta))-kt\theta(32(1-\delta)-4k(13-\delta)(1-\delta)\theta+4k^2(6-\delta)(1-\delta)\theta^2-k^3(2-\delta)^2\theta^3))^2}$$

,

$$e^{ER}=$$
$$\Big(k(1-\delta)\theta\big(P_e\big(-16+20\delta+26k\theta-4k(9-\delta)\delta\theta-k^2(4-\delta)(3-4\delta)\theta^2+k^3(2-\delta)(1-\delta)\theta^3\big)+(1-\delta)\eta(1-k\theta)(\delta(6-k\theta)(2-k\theta)-2(8-k\theta(5-k\theta)))\big)+$$
$$e_0P_e\big(2P_e(1-k\theta)(8-k\theta(5-k\theta)-\delta(4-k\theta)(2-k\theta))-(1-\delta)\eta\big(k\delta^2\theta(2-k\theta)^2+2\delta(4-k\theta)(2-k\theta)(1-k\theta)-2(1-k\theta)(8-k\theta(5-k\theta))\big)\big)\Big)\Big/$$
$$\Big(2P_e^2(1-k\theta)(8-k\theta(5-k\theta)-\delta(4-k\theta)(2-k\theta))+(1-\delta)\big(2(1-\delta)\eta^2(1-k\theta)\big(8(1-\delta)-k(5-6\delta)\theta+k^2(1-\delta)\theta^2\big)-kt\theta\big(32(1-\delta)-4k(13-\delta)(1-\delta)\theta+4k^2(6-\delta)(1-\delta)\theta^2-k^3(2-\delta)^2\theta^3\big)\big)-$$
$$2P_e(1-\delta)\eta\big(k\delta^2\theta(2-k\theta)^2+2\delta(4-k\theta)(2-k\theta)(1-k\theta)-2(1-k\theta)(8-k\theta(5-k\theta))\big)\Big);$$

$$w^{ER}=$$
$$\Big((1-\delta)(1-k\theta)\big(P_e^2\big(-8-k\theta(2-k\theta)(1-4\delta-k(1-\delta)\theta)-e_0\eta\big(8(2-\delta)-2k(5-3\delta)\theta+k^2(2-\delta)\theta^2\big)\big)+(-1+\delta)\big((1-\delta)\eta^2(4-k\theta)(2-k\theta)(1-k\theta)-2kt\theta(8-k\theta(2(3+\delta)-k(2+\delta)\theta))\big)+$$
$$P_e\big(e_0(1-\delta)\eta^2\big(-8(2-\delta)+2k(5-3\delta)\theta-k^2(2-\delta)\theta^2\big)+e_0kt\theta(16-2k\theta(5-k\theta)-\delta(4-k\theta)(2-k\theta))-(1-\delta)\eta(16-k\theta(12-k\theta(4-\delta(2-k\theta))))\big)\big)\Big)\Big/$$
$$\Big(2P_e(1-\delta)\eta\big(k\delta^2\theta(2-k\theta)^2+2\delta(4-k\theta)(2-k\theta)(1-k\theta)-2(1-k\theta)(8-k\theta(5-k\theta))\big)-$$
$$\big(2P_e^2(1-k\theta)(8-k\theta(5-k\theta)-\delta(4-k\theta)(2-k\theta))+(1-\delta)\big(2(1-\delta)\eta^2(1-k\theta)\big(8(1-\delta)-k(5-6\delta)\theta+k^2(1-\delta)\theta^2\big)-kt\theta\big(32(1-\delta)-4k(13-\delta)(1-\delta)\theta+4k^2(6-\delta)(1-\delta)\theta^2-k^3(2-\delta)^2\theta^3\big)\big)\big)\Big);$$

$$P_b^{ER}=$$
$$\Big((1-k\theta)\big(P_e^2\big(4(1-\delta)(3+e_0(4-\delta)\eta)-2k\big(1-2\delta^2+e_0(5-2(4-\delta)\delta)\eta\big)\theta-k^2(1-\delta)(3-4\delta-e_0(2-\delta)\eta)\theta^2+k^3(1-\delta)^2\theta^3\big)-$$
$$(1-\delta)\big(2kt\theta(2-k\theta)(6-2k\theta-3\delta(2-k\theta))-(1-\delta)\eta^2(1-k\theta)(12-k\theta(6-k\theta)-\delta(6-k\theta)(2-k\theta))\big)+$$
$$P_e\big(e_0(1-\delta)\eta^2\big(4(4-\delta)(1-\delta)-2k(5-2(4-\delta)\delta)\theta+k^2(2-\delta)(1-\delta)\theta^2\big)-e_0kt\theta\big(4(4-\delta)(1-\delta)-2k(5-2(4-\delta)\delta)\theta+k^2(2-\delta)(1-\delta)\theta^2\big)+(1-\delta)\eta(2-k\theta)(12-4k\theta-\delta(2-k\theta)(6-k(1-\delta)\theta))\big)\big)\Big)\Big/$$
$$\Big(2P_e^2(1-k\theta)(8-k\theta(5-k\theta)-\delta(4-k\theta)(2-k\theta))+(1-\delta)\big(2(1-\delta)\eta^2(1-k\theta)\big(8(1-\delta)-k(5-6\delta)\theta+k^2(1-\delta)\theta^2\big)-kt\theta\big(32(1-\delta)-4k(13-\delta)(1-\delta)\theta+4k^2(6-\delta)(1-\delta)\theta^2-k^3(2-\delta)^2\theta^3\big)\big)-$$
$$2P_e(1-\delta)\eta\big(k\delta^2\theta(2-k\theta)^2+2\delta(4-k\theta)(2-k\theta)(1-k\theta)-2(1-k\theta)(8-k\theta(5-k\theta))\big)\Big);$$

$$P_m^{ER}=$$
$$\Big((1-k\theta)\big(k\theta\big(P_e(1-\delta)\eta(20-4k\theta(4-k\theta)-\delta(4-k\theta)(2-k\theta))+P_e^2(18-k\theta(13-3k\theta)-\delta(2-k\theta)(8-3k\theta))+(1-\delta)\big((1-\delta)\eta^2(2-k\theta)(1-k\theta)-2kt\theta\big(10-6\delta-k(8-5\delta)\theta+k^2(2-\delta)\theta^2\big)\big)\big)-$$
$$e_0P_e(\eta(P_e+\eta-\delta\eta)-kt\theta)(\delta(2-k\theta)(8-3k\theta)-2(8-k\theta(5-k\theta)))\big)\Big)\Big/$$
$$\Big(2P_e^2(1-k\theta)(8-k\theta(5-k\theta)-\delta(4-k\theta)(2-k\theta))+(1-\delta)\big(2(1-\delta)\eta^2(1-k\theta)\big(8(1-\delta)-k(5-6\delta)\theta+k^2(1-\delta)\theta^2\big)-kt\theta\big(32(1-\delta)-4k(13-\delta)(1-\delta)\theta+4k^2(6-\delta)(1-\delta)\theta^2-k^3(2-\delta)^2\theta^3\big)\big)-$$
$$2P_e(1-\delta)\eta\big(k\delta^2\theta(2-k\theta)^2+2\delta(4-k\theta)(2-k\theta)(1-k\theta)-2(1-k\theta)(8-k\theta(5-k\theta))\big)\Big);$$

```
Dm =
 - ((-Pe^2 (-2 + k θ) (3 - 2 δ + k (-1 + δ) θ) (1 + e0 δ η + k (-1 + δ) θ) + (-1 + δ) (-((-1 + δ) η^2 (-3 + k θ) (-2 + k θ) (-1 + k θ)) + 2 k t θ (-2 + 2 k θ + δ (-2 + k θ) (-3 + 2 k θ))) +
      Pe (-2 (-1 + δ) η (-2 + k θ) (-3 + δ + k (4 - 3 δ) θ + k^2 (-1 + δ) θ^2) + e0 (-((-1 + δ) δ η^2 (-3 + k θ) (-2 + k θ)) + t (k δ^2 θ (-2 + k θ)^2 + 2 (-1 + k θ) (8 + k θ (-5 + k θ)) - δ (-2 + k θ) (8 + k θ (-13 + 3 k θ)))))) /
    (2 Pe^2 (-1 + k θ) (-8 + k θ (5 - k θ) + δ (-4 + k θ) (-2 + k θ)) + (-1 + δ) (2 (-1 + δ) η^2 (-1 + k θ) (8 (-1 + δ) + k (5 - 6 δ) θ + k^2 (-1 + δ) θ^2) + k t θ (-32 (-1 + δ) - 4 k (-13 + δ) (-1 + δ) θ + 4 k^2 (-6 + δ) (-1 + δ) θ^2 - k^3 (-2 + δ)^2 θ^3)) +
      2 Pe (-1 + δ) η (k δ^2 θ (-2 + k θ)^2 - 2 δ (-4 + k θ) (-2 + k θ) (-1 + k θ) + 2 (-1 + k θ) (8 + k θ (-5 + k θ)))));
Db = ((2 - k θ) (2 - δ (2 - k θ)) (Pe (1 - δ) η (2 + e0 δ η) - k Pe (e0 t δ + (2 - δ) (1 - δ) η) θ + Pe^2 (1 + e0 δ η - k (1 - δ) θ) + (1 - δ) ((1 - δ) η^2 (1 - k θ) - k t θ (2 - k (2 - δ) θ)))) /
   (2 Pe^2 (1 - k θ) (8 - k θ (5 - k θ) - δ (4 - k θ) (2 - k θ)) + (1 - δ) (2 (1 - δ) η^2 (1 - k θ) (8 (1 - δ) - k (5 - 6 δ) θ + k^2 (1 - δ) θ^2) - k t θ (32 (1 - δ) - 4 k (13 - δ) (1 - δ) θ + 4 k^2 (6 - δ) (1 - δ) θ^2 - k^3 (2 - δ)^2 θ^3)) -
    2 Pe (1 - δ) η (k δ^2 θ (2 - k θ)^2 + 2 δ (4 - k θ) (2 - k θ) (1 - k θ) - 2 (1 - k θ) (8 - k θ (5 - k θ))));
```

Proof of Lemma 2 Given the profit ${\pi_m}^{NR}$ showed in Eq 1, we have $\frac{\partial^2 {\pi_b}^{NR}}{\partial (p_b^{NR})^2} = -2.$, thus ${\pi_b}^{NR}$ is strictly concave in p_b^{NR}. Using the FOC yields $p_b^{NR}(w^{NR}, e^{NR}) = \frac{1}{2}(1 + w + e\eta)$. (A1)

Substituting Eq. (A1) into π_m^{NR}, we have, when $\frac{\partial^2 \pi_m^{NR}}{\partial (w^{NR})^2} = -1$ and $\frac{\partial^2 \pi_m^{NR}}{\partial (e^{NR})^2} = \eta p_e - t < 0$. Let H be a Hessian of π_m^{NR} is $H = \begin{bmatrix} \eta p_e - t & -\frac{1}{2}(S - \eta) \\ -\frac{1}{2}(S - \eta) & -1 \end{bmatrix} = t - \frac{1}{4}(p_e + \eta)^2 > 0$. H is a negative definite.

Hence, π_m^{NR} is concave in w^{NR} and e^{NR}, we derive the following by using the FOCs $w^{NR} = \frac{p_e\left(p_e + \eta + e_o p_e \eta - e_o(2t - \eta^2)\right) - 2t}{(p_e + \eta)^2 - 4t}$, $e^{NR} = \frac{(e_o p_e - 1)(p_e + \eta)}{(p_e + \eta)^2 - 4t}$. Substituting w^{NR} and e^{NR} into (A1) yields the equilibrium $p_b^{NR} = \frac{p_e(p_e + \eta + e_o p_e \eta - e_o(t - \eta^2)) - 3t}{(p_e + \eta)^2 - 4t}$.

Proof of Lemma 3 $\frac{\partial e^{NR}}{\partial \eta} = \frac{(1 - e_o p_e)(4t + (p_e + \eta)^2)}{(4t - (p_e + \eta)^2)^2} > 0, \frac{\partial w^{NR}}{\partial \eta} = \frac{(1 - e_o p_e)(4t\eta - {p_e}^3 - 2{p_e}^2 \eta - p_e \eta^2)}{(4t - (p_e + \eta)^2)^2} > 0$

$\frac{\partial {D_b}^{NR}}{\partial \eta} = \frac{2(1 - e_o p_e)t(p_e + \eta)}{(4t - (p_e + \eta)^2)^2} > 0$, $\frac{\partial {\pi_m}^{NR}}{\partial \eta} = \frac{(1 - e_o p_e)^2 t(p_e + \eta)}{(4t - (p_e + \eta)^2)^2} > 0$, $\frac{\partial {\pi_b}^{NR}}{\partial \eta} = \frac{4(1 - e_o p_e)^2 t^2 (p_e + \eta)}{(4t - (p_e + \eta)^2)^3} > 0$.

Proof of Lemma 4 Given the profit π_m^{EU} showed in Eq. , we have $\frac{\partial^2 \pi_m^{EU}}{\partial (p_m^{EU})^2} = -\frac{2(1 - \delta)}{k\theta(1 - k\theta)}$, Because $0 \le k\theta < 1$, thus π_m^{EU} is strictly concave in p_m^{EU}. Using the FOC yields $p_m^{EU}(w, e, p_b) = \frac{1}{2}\left[\frac{kw\theta}{1 - \delta} + e(\eta - k\eta\theta) + k\theta p_b\right]$.

Substituting $p_m^{EU}(w, e, p_b)$ into $\pi_b^{EU}(w, e, p_b)$, we have $\frac{\partial^2 \pi_b^{EU}}{\partial ({p_b}^{EU})^2} = -1 + \frac{1}{-1 + k\theta} < 0$. Thus, we derive the following by using the FOCs, $p_b^{EU}(w^{EU}, e^{EU}) = \frac{(1 - \delta)(2 + e\eta)(1 - k\theta) + w(2 - \delta(2 - k\theta))}{2(1 - \delta)(2 - k\theta)}$. Then, substituting $p_b^{EU}(w, e)$ and $p_m^{EU}(w, e, p_b)$ into $\pi_m^{EU}(w, e)$ we have $\frac{\partial^2 \pi_m^{EU}}{\partial (e^{EU})^2} = -t + \frac{(1 - \delta)\eta^2(4 - k\theta)^2(1 - k\theta)}{8k\theta(2 - k\theta)^2} < 0$, $\frac{\partial^2 \pi_m^{EU}}{\partial (w^{EU})^2} = \frac{-32(1 - \delta) + 4k(13 - \delta)(1 - \delta)\theta - 4k^2(6 - \delta)(1 - \delta)\theta^2 + k^3(2 - \delta)^2\theta^3}{8(1 - \delta)(2 - k\theta)^2(1 - k\theta)} < 0$. Since the dual channel demand constraint are the linear function of w^{EU}, e^{EU}, $\pi_m^{EU}(w^{EU}, e^{EU})$ are jointly concave in w^{EU}, e^{EU}, thus the Kuhn–Tucker conditions are necessary and sufficient for solving problem of optimal profit. The K-T conditions are as follows:

$\frac{\partial \pi_m{}^{EU}}{\partial e^{EU}} + \lambda_1 \frac{\partial\left(p_b^{EU} - \frac{p_m^{EU} - (1-k\theta)\eta e^{EU}}{k\theta}\right)}{\partial e^{EU}} + \lambda_2 = 0;$

$\frac{\partial \pi_m{}^{EU}}{\partial w^{EU}} + \lambda_1 \frac{\partial\left(p_b^{EU} - \frac{p_m^{EU} - (1-k\theta)\eta e^{EU}}{k\theta}\right)}{\partial w^{EU}} + \lambda_2 = 0;$

$\lambda_1 \frac{\partial\left(p_b^{EU} - \frac{p_m^{EU} - (1-k\theta)\eta e^{EU}}{k\theta}\right)}{\partial w^{EU}} = 0;$

$\lambda_2 \frac{\partial(1 + p_m^{EU} - k\theta - p_b^{EU})}{\partial w^{EU}} = 0;$

where $\lambda_1 \geq 0$, $\lambda_2 \geq 0$, $\lambda_3 \geq 0$ and $\lambda_4 \geq 0$ are the Lagrange multipliers.

According to the K-T conditions, we can get the following situations:

(1) $e^{EU} = e^{EU\#} = \frac{k(-1+\delta)\eta\theta(-1+k\theta)(\delta(-6+k\theta)(-2+k\theta)-2(8+k\theta(-5+k\theta)))}{kt\theta(4k(13-\delta)(1-\delta)\theta-32(1-\delta)-4k^2(6-\delta)(1-\delta)\theta^2+k^3(2-\delta)^2\theta^3)-2(1-\delta)\eta^2(1-k\theta)(k\theta(5-k\theta)-8+\delta(4-k\theta)(2-k\theta))}$,

$w^{EU} = w^{EU\#} = \frac{(-1+\delta)(-1+k\theta)((-1+\delta)\eta^2(-4+k\theta)(-2+k\theta)(-1+k\theta)+2kt\theta(-8+k\theta(2(3+\delta)-k(2+\delta)\theta)))}{kt\theta(4k(13-\delta)(1-\delta)\theta-32(1-\delta)-4k^2(6-\delta)(1-\delta)\theta^2+k^3(2-\delta)^2\theta^3)-2(1-\delta)\eta^2(1-k\theta)(k\theta(5-k\theta)-8+\delta(4-k\theta)(2-k\theta))}$ for $\frac{p_m-(1-k\theta)\eta e}{k\theta} < p_b \leq 1 + p_m - k\theta$, when $\lambda_1 = 0$ and $\lambda_2 = 0$.

(2) When $\lambda_1 = 0$, $\lambda_2 = -\frac{2(-1+\delta)(2+\delta(-2+k\theta))(-((-1+\delta)\eta^2(-1+k\theta))+kt\theta(2+k(-2+\delta)\theta))}{(-2+k\theta)(-2(-1+\delta)^2\eta^2(-1+k\theta)(-1+\delta+k\theta)+kt\theta(2-2k\theta+\delta(-2+k\theta))^2)} < 0$, therefore the solution is not the optimal one.

(3) When $\lambda_1 = \frac{2k(-1+\delta)\theta((-1+\delta)\eta^2(-3+k\theta)(-2+k\theta)(-1+k\theta)-2kt\theta(-2+2k\theta+\delta(-2+k\theta)(-3+2k\theta)))}{-2(-1+\delta)\eta^2(-4+k\theta)(-2+k\theta)(-1+k\theta)(-1+\delta+k\theta)+k^2t\theta^2(-2(1+\delta)+k(2+\delta)\theta)^2} < 0$, $\lambda_2 = 0$, therefore the solution is not the optimal one.

(4) When $\lambda_1 = -\frac{k\theta\left(((1-\delta)(1-\delta-k\theta)) - \frac{kt\delta\theta(-2+2k\theta+\delta(2-k\theta))}{\eta^2(1-k\theta)}\right)}{(1-\delta-k\theta)^2}$, $\lambda_2 = -\frac{((1-\delta)(1-\delta-k\theta)) + \frac{k^2t\delta\theta^2(2(1-\delta)-k(2+\delta)\theta)}{\eta^2(2-k\theta)(1-k\theta)}}{(1-\delta-k\theta)^2}$, in this case we can also get $\lambda_1 < 0$, $\lambda_2 < 0$, therefore the solution is not the optimal one.

Substuting $e^{EU\#}$, $p_m^{EU\#}$ and $p_b^{EU\#}$ into constraint $\frac{p_m-(1-k\theta)\eta e}{k\theta} < p_b \leq 1 + p_m - k\theta$, We can get threshold t_1, when $t > t_1$, dual channel case exists, it means that manufacturer always chooses to launch FB products, when $t < t_1$, brand product exit the market, this result tends to be impossible, so omit it.

Then, substituting w^{EU} and e^{EU} into (A1) yields the equilibrium as follows.

$D_m{}^{EU} = D_m{}^{EU\#} = \frac{(2-k\theta)(2-\delta(2-k\theta))(kt\theta(2-k(2-\delta)\theta)-(1-\delta)\eta^2(1-k\theta))}{kt\theta(32(1-\delta)-4k(13-\delta)(1-\delta)\theta+4k^2(6-\delta)(1-\delta)\theta^2-k^3(2-\delta)^2\theta^3)-2(1-\delta)\eta^2(1-k\theta)(8-k\theta(5-k\theta)-\delta(4-k\theta)(2-k\theta))}$;

$D_b{}^{EU} = D_b{}^{EU\#} =$

$$\frac{(1-\delta)\eta^2(3-k\theta)(2-k\theta)(1-k\theta)+2kt\theta(2-2k\theta-\delta(2-k\theta)(3-2k\theta))}{kt\theta(32(1-\delta)-4k(13-\delta)(1-\delta)\theta+4k^2(6-\delta)(1-\delta)\theta^2-k^3(2-\delta)^2\theta^3)-2(1-\delta)\eta^2(1-k\theta)(8-k\theta(5-k\theta)-\delta(4-k\theta)(2-k\theta))};$$

Substituting ${e}^{EU\#}$, ${w}^{EU\#}$, ${D_m}^{EU\#}$ and ${D_b}^{EU\#}$ into π_b^{EU}, π_m^{EU} and π_p^{EU},we have

$\pi_b^{EU} = \pi_b^{EU\#} =$

$$\frac{2(2-k\theta)(1-k\theta)(2-\delta(2-k\theta))^2((1-\delta)\eta^2(1-k\theta)-kt\theta(2-k(2-\delta)\theta))^2}{(kt\theta(32(1-\delta)-4k(13-\delta)(1-\delta)\theta+4k^2(6-\delta)(1-\delta)\theta^2-k^3(2-\delta)^2\theta^3)+2(1-\delta)\eta^2(1-k\theta)(-8+k\theta(5-k\theta)+\delta(4-k\theta)(2-k\theta)))^2};$$

$\pi_m^{EU} = \pi_m^{EU\#} =$

$$\frac{(1-\delta)(1-k\theta)((1-\delta)\eta^2(2-k\theta)^2(1-k\theta)-4kt\theta(2-k\delta\theta(2-k\theta)))}{4(1-\delta)\eta^2(1-k\theta)(8-k\theta(5-k\theta)-\delta(4-k\theta)(2-k\theta))-2kt\theta(32(1-\delta)-4k(13-\delta)(1-\delta)\theta+4k^2(6-\delta)(1-\delta)\theta^2-k^3(2-\delta)^2\theta^3)};$$

$\pi_p^{EU} = \pi_p^{EU\#} =$

$$\frac{k\delta\theta(1-k\theta)((1-\delta)\eta^2(2-k\theta)(1-k\theta)+2kt\theta(6\delta+k(8-5\delta)\theta-k^2(2-\delta)\theta^2-10))(2kt\theta(2k\theta-2+\delta(2-k\theta)(3-2k\theta))-(1-\delta)\eta^2(3-k\theta)(2-k\theta)(1-k\theta))}{(2(1-\delta)\eta^2(1-k\theta)(8-k\theta(5-k\theta)-\delta(4-k\theta)(2-k\theta))-kt\theta(32(1-\delta)-4k(13-\delta)(1-\delta)\theta+4k^2(6-\delta)(1-\delta)\theta^2-k^3(2-\delta)^2\theta^3))^2}$$

.

Proof of Lemma 5.

$\frac{\partial e^{EU}}{\partial \eta} =$

$$\frac{k(1-\delta)\theta(1-k\theta)(2(8-k\theta(5-k\theta))-\delta(6-k\theta)(2-k\theta))(kt\theta(32(1-\delta)-4k(13-\delta)(1-\delta)\theta+4k^2(6-\delta)(1-\delta)\theta^2-k^3(2-\delta)^2\theta^3)+2(1-\delta)\eta^2(1-k\theta)(8-k\theta(5-k\theta)-\delta(\text{[illegible]}}{(kt\theta(32(1-\delta)-4k(13-\delta)(1-\delta)\theta+4k^2(6-\delta)(1-\delta)\theta^2-k^3(2-\delta)^2\theta^3)-2(1-\delta)\eta^2(1-k\theta)(8-k\theta(5-k\theta)-\delta(4-k\theta)(2-k\theta)))^2}$$

> 0 ; $\frac{\partial e^{EU}}{\partial t} =$

$$\frac{k^2(1-\delta)\eta\theta^2(1-k\theta)(32(1-\delta)-4k(13-\delta)(1-\delta)\theta+4k^2(6-\delta)(1-\delta)\theta^2-k^3(2-\delta)^2\theta^3)(\delta(6-k\theta)(2-k\theta)-2(8-k\theta(5-k\theta)))}{(kt\theta(32(1-\delta)-4k(13-\delta)(1-\delta)\theta+4k^2(6-\delta)(1-\delta)\theta^2-k^3(2-\delta)^2\theta^3)-2(1-\delta)\eta^2(1-k\theta)(8-k\theta(5-k\theta)-\delta(4-k\theta)(2-k\theta)))^2} <$$

0 ; $\frac{\partial w^{EU}}{\partial \eta} =$

$$\frac{2k^2t(1-\delta)^2\eta\theta^2(1-k\theta)^2(8(2-\delta)-2k(5-3\delta)\theta+k^2(2-\delta)\theta^2)(2(8-k\theta(5-k\theta))-\delta(6-k\theta)(2-k\theta))}{(kt\theta(32(1-\delta)-4k(13-\delta)(1-\delta)\theta+4k^2(6-\delta)(1-\delta)\theta^2-k^3(2-\delta)^2\theta^3)-2(1-\delta)\eta^2(1-k\theta)(8-k\theta(5-k\theta)-\delta(4-k\theta)(2-k\theta)))^2} >$$

0 . $\frac{\partial w^{EU}}{\partial t} =$

$$\frac{k^2(1-\delta)^2\eta^2\theta^2(1-k\theta)^2(8(2-\delta)-2k(5-3\delta)\theta+k^2(2-\delta)\theta^2)(\delta(6-k\theta)(2-k\theta)-2(8-k\theta(5-k\theta)))}{(kt\theta(32(1-\delta)-4k(13-\delta)(1-\delta)\theta+4k^2(6-\delta)(1-\delta)\theta^2-k^3(2-\delta)^2\theta^3)-2(1-\delta)\eta^2(1-k\theta)(8-k\theta(5-k\theta)-\delta(4-k\theta)(2-k\theta)))^2} <$$

0 ; $\frac{\partial {D_b}^{EU}}{\partial \eta} =$

$$\frac{2kt(1-\delta)\eta\theta(1-k\theta)(\delta(6-k\theta)(2-k\theta)-2(8-k\theta(5-k\theta)))(\delta(2-k\theta)(8-k\theta(7-k\theta))-2(1-k\theta)(8-k\theta(5-k\theta)))}{(kt\theta(32(1-\delta)-4k(13-\delta)(1-\delta)\theta+4k^2(6-\delta)(1-\delta)\theta^2-k^3(2-\delta)^2\theta^3)-2(1-\delta)\eta^2(1-k\theta)(8-k\theta(5-k\theta)-\delta(4-k\theta)(2-k\theta)))^2} <$$

0 ; $\frac{\partial {D_b}^{EU}}{\partial t} =$

$$\frac{k(1-\delta)\eta^2\theta(1-k\theta)(\delta(6-k\theta)(2-k\theta)-2(8-k\theta(5-k\theta)))(2(1-k\theta)(8-k\theta(5-k\theta))-\delta(2-k\theta)(8-k\theta(7-k\theta)))}{(kt\theta(32(1-\delta)-4k(13-\delta)(1-\delta)\theta+4k^2(6-\delta)(1-\delta)\theta^2-k^3(2-\delta)^2\theta^3)-2(1-\delta)\eta^2(1-k\theta)(8-k\theta(5-k\theta)-\delta(4-k\theta)(2-k\theta)))^2} <$$

0 ; $\frac{\partial {D_m}^{EU}}{\partial \eta} =$

$$\frac{2k^2t(1-\delta)\delta\eta\theta^2(2-k\theta)(1-k\theta)(2-\delta(2-k\theta))(2(8-k\theta(5-k\theta))-\delta(6-k\theta)(2-k\theta))}{(kt\theta(32(1-\delta)-4k(13-\delta)(1-\delta)\theta+4k^2(6-\delta)(1-\delta)\theta^2-k^3(2-\delta)^2\theta^3)-2(1-\delta)\eta^2(1-k\theta)(8-k\theta(5-k\theta)-\delta(4-k\theta)(2-k\theta)))^2} >$$

0 ; $\frac{\partial D_m{}^{EU}}{\partial t} = \frac{k^2(1-\delta)\delta\eta^2\theta^2(2-k\theta)(1-k\theta)(2-\delta(2-k\theta))(\delta(6-k\theta)(2-k\theta)-2(8-k\theta(5-k\theta)))}{(kt\theta(32(1-\delta)-4k(13-\delta)(1-\delta)\theta+4k^2(6-\delta)(1-\delta)\theta^2-k^3(2-\delta)^2\theta^3)-2(1-\delta)\eta^2(1-k\theta)(8-k\theta(5-k\theta)-\delta(4-k\theta)(2-k\theta)))^2} < 0$;

$\frac{\partial \pi_m^{EU}}{\partial \eta} = \frac{k^2t(1-\delta)^2\eta\theta^2(1-k\theta)^2(\delta(6-k\theta)(2-k\theta)-2(8-k\theta(5-k\theta)))^2}{(kt\theta(32(1-\delta)-4k(13-\delta)(1-\delta)\theta+4k^2(6-\delta)(1-\delta)\theta^2-k^3(2-\delta)^2\theta^3)-2(1-\delta)\eta^2(1-k\theta)(8-k\theta(5-k\theta)-\delta(4-k\theta)(2-k\theta)))^2} > 0$;

$\frac{\partial \pi_m^{EU}}{\partial t} = -\frac{k^2(1-\delta)^2\eta^2\theta^2(1-k\theta)^2\left(\delta(6-k\theta)(2-k\theta)-2\left(8-k\theta(5-k\theta)\right)\right)^2}{2\left(kt\theta(32(-1+\delta)+4k(13-\delta)(1-\delta)\theta-4k^2(6-\delta)(1-\delta)\theta^2+k^3(2-\delta)^2\theta^3)-2(1-\delta)\eta^2(1-k\theta)\left(-8+k\theta(5-k\theta)+\delta(4-k\theta)(2-k\theta)\right)\right)^2} < 0$;

$\frac{\partial \pi_b^{EU}}{\partial \eta} = \frac{8k^2t(1-\delta)\delta\eta\theta^2(2-k\theta)(1-k\theta)^2(2-\delta(2-k\theta))^2(((1-\delta)\eta^2(1-k\theta))-kt\theta(2-k(2-\delta)\theta))(\delta(6-k\theta)(2-k\theta)-2(8-k\theta(5-k\theta)))}{(kt\theta(32(1-\delta)-4k(13-\delta)(1-\delta)\theta+4k^2(6-\delta)(1-\delta)\theta^2-k^3(2-\delta)^2\theta^3)-2(1-\delta)\eta^2(1-k\theta)(8-k\theta(5-k\theta)-\delta(4-k\theta)(2-k\theta)))^3} > 0$; $\frac{\partial \pi_b^{EU}}{\partial t} = \frac{4k^2(1-\delta)\delta\eta^2\theta^2(2-k\theta)(1-k\theta)^2(2-\delta(2-k\theta))^2(((1-\delta)\eta^2(1-k\theta))-kt\theta(2-k(2-\delta)\theta))(2(8-k\theta(5-k\theta))-\delta(6-k\theta)(2-k\theta))}{(kt\theta(32(1-\delta)-4k(13-\delta)(1-\delta)\theta+4k^2(6-\delta)(1-\delta)\theta^2-k^3(2-\delta)^2\theta^3)-2(1-\delta)\eta^2(1-k\theta)(8-k\theta(5-k\theta)-\delta(4-k\theta)(2-k\theta)))^3} > 0$;

$\frac{\partial \pi_p^{EU}}{\partial \eta} = \frac{4k^2t(1-\delta)\delta\eta\theta^2(1-k\theta)^2\left(\delta(6-k\theta)(2-k\theta)-2\left(8-k\theta(5-k\theta)\right)\right)}{(kt\theta(32(1-\delta)-4k(13-\delta)(1-\delta)\theta+4k^2(6-\delta)(1-\delta)\theta^2-k^3(2-\delta)^2\theta^3)-2(1-\delta)\eta^2(1-k\theta)(8-k\theta(5-k\theta)-\delta(4-k\theta)(2-k\theta)))^2} * (kt\theta(4\delta(2-k\theta)(6-k\theta(6-k\theta))(8-k\theta(5-k\theta))-4(1-k\theta)(8-k\theta(5-k\theta))(6-k\theta(4-k\theta))-\delta^2(2-k\theta)^2(48-k\theta(54-k\theta(16-k\theta))))-(1-\delta)\eta^2(2-k\theta)(1-k\theta)(8-k\theta(5-k\theta))(2-\delta(2-k\theta)))) < 0$;

$\frac{\partial \pi_p^{EU}}{\partial t} = \frac{2k^2(1-\delta)\delta\eta^2\theta^2(1-k\theta)^2(2(8-k\theta(5-k\theta))-\delta(6-k\theta)(2-k\theta))}{(kt\theta(32(1-\delta)-4k(13-\delta)(1-\delta)\theta+4k^2(6-\delta)(1-\delta)\theta^2-k^3(2-\delta)^2\theta^3)-2(1-\delta)\eta^2(1-k\theta)(8-k\theta(5-k\theta)-\delta(4-k\theta)(2-k\theta)))^3} * (kt\theta(4\delta(2-k\theta)(6-k\theta(6-k\theta))(8-k\theta(5-k\theta))-4(1-k\theta)(8-k\theta(5-k\theta))(6-k\theta(4-k\theta))-\delta^2(2-k\theta)^2(48-k\theta(54-k\theta(16-k\theta))))-(1-\delta)\eta^2(2-k\theta)(1-k\theta)(8-k\theta(5-k\theta))(2-\delta(2-k\theta)))) < 0$.

Proof of Lemma 6. Given the profit π_m^{ER} showed in Eq. , we have $\frac{\partial^2 \pi_m^{ER}}{\partial (p_m^{EU})^2} = -\frac{2(1-\delta)}{k\theta(1-k\theta)}$, Because $0 \le k\theta < 1$, thus π_m^{EU} is strictly concave in p_m^{EU}. Using the FOC yields $p_m^{EU}(w, e, p_b) = \frac{1}{2}\left[\frac{e_0p_e-k(e_0p_e-w)\theta-e(p_e-(1-\delta)\eta)(1-k\theta)+k(1-\delta)\theta p_b}{(1-\delta)}\right]$.

Substituting $p_m^{ER}(w,e,p_b)$ into $\pi_b^{ER}(w,e,p_b)$, we have $\frac{\partial^2 \pi_b^{ER}}{\partial({p_b}^{ER})^2} = -1 + \frac{1}{-1+k\theta} < 0$. Thus, we derive the following by using the FOC, $p_b^{EU}(w^{EU},e^{EU}) = \frac{2(1+w)(1-\delta)-k(2-(2+w)\delta)\theta-e(P-(1-\delta)\eta)(1-k\theta)+j(P-kP\theta)}{2(1-\delta)(2-k\theta)}$. Substituting $p_b^{EU}(w^{EU},e^{EU})$ and $p_m^{ER}(w,e,p_b)$ into π_m^{ER}, we have $\frac{\partial^2 \pi_m^{ER}}{\partial(e^{ER})^2} = -t + \frac{(1-\delta)\eta^2(4-k\theta)^2(1-k\theta)}{8k\theta(2-k\theta)^2} < 0$, $\frac{\partial^2 \pi_m^{ER}}{\partial(w^{ER})^2} = \frac{-32(1-\delta)+4k(13-\delta)(1-\delta)\theta-4k^2(6-\delta)(1-\delta)\theta^2+k^3(2-\delta)^2\theta^3}{8(1-\delta)(2-k\theta)^2(1-k\theta)} < 0$. Since the dual channel demand constraint are the linear function of w^{ER}, e^{ER}, $\pi_m^{EU}(w^{ER},e^{ER})$ are jointly concave in w^{ER}, e^{ER}, thus the Kuhn–Tucker conditions are necessary and sufficient for solving problem of optimal profit. The K-T conditions are as follows:

$$\frac{\partial {\pi_m}^{ER}}{\partial e^{ER}} + \lambda_1 \frac{\partial\left(p_b^{ER} - \frac{p_m^{ER}-(1-k\theta)\eta e^{ER}}{k\theta}\right)}{\partial e^{ER}} + \lambda_2 = 0;$$

$$\frac{\partial {\pi_m}^{ER}}{\partial w^{ER}} + \lambda_1 \frac{\partial\left(p_b^{ER} - \frac{p_m^{ER}-(1-k\theta)\eta e^{ER}}{k\theta}\right)}{\partial w^{EU}} + \lambda_2 = 0;$$

$$\lambda_1 \frac{\partial\left(p_b^{ER} - \frac{p_m^{ER}-(1-k\theta)\eta e^{ER}}{k\theta}\right)}{\partial w^{EU}} = 0;$$

$$\lambda_2 \frac{\partial\left(1+p_m^{ER}-k\theta-p_b^{ER}\right)}{\partial w^{ER}} = 0;$$

According to the Kuhn-Tucker conditions, we can get the following situations

(1) $e^{ER} = e^{ER\#1}$, $w^{ER} = w^{ER\#}$; for $\frac{p_m-(1-k\theta)\eta e}{k\theta} < p_b \leq 1 + p_m - k\theta$, when $\lambda_1 = 0$ and $\lambda_2 = 0$.

(2) When $\lambda_1 \neq 0$ and $\lambda_2 \neq 0$, then in this case we can get $\lambda_1 > 0$ however $\lambda_2 < 0$, thus, this solution is not the optimal one.

(3) When $\lambda_1 = 0$ and $\lambda_2 \neq 0$, in this case $\lambda_2 < 0$, thus, this solution is not the optimal one.

(4) When $\lambda_1 \neq 0$, $\lambda_2 = 0$, in this case $\lambda_1 > 0$, then $e^{ER} = e^{ER\#2} =$

$$\frac{(-2+k\theta)(k\theta(2(-1+3\delta)(P+\eta-\delta\eta)+k(P(-4+\delta)(-1+2\delta)+(-1+\delta)(-4+7\delta)\eta)\theta-k^2(-2+\delta)(-1+\delta)(P+\eta)\theta^2)+jP(-8(-1+\delta)\eta+2P(-1+k\theta)(-4+k\theta(5-2\delta+k(- \text{[illegible]}}{2(-1+\delta)\eta^2(-4+k\theta)(-2+k\theta)(-1+k\theta)(-1+\delta+k\theta)-k^2t\theta^2(-2(1+\delta)+k(2+\delta)\theta)^2+2P\eta(-2+k\theta)(k\delta^2\theta(-2+k\theta)+2\delta(-2+k\theta)^2(-1+k\theta)-2(-4+k\theta)(-1+ \text{[illegible]}}$$

;

$w^{ER} = w^{ER\#2} =$

$((-1+k\theta)(P^2(-2+k\theta)(-4+4j(-2+\delta)\eta+k(3+j(10-7\delta)\eta)\theta+k^2(-1+\delta)(-1+2j\eta)\theta^2)+(-1+\delta)(-((-1$ [illegible]

;

In this case, we can get ${D_m}^{ER\#2} = 0$, which means although factory open the online channel, but there is no factory product in the market, customer can only buy brand products from retailer channel.

The constant parameters X_1 to X_{10} are defined as below to simplify the presentation of equilibrium results:

Proposition 1 Subtracting π_m^{NU} from π_m^{EU} yields $\pi_m^{EU} - \pi_m^{NU} = \frac{A_1 {e_0}^2 + B_1 e_0 + C_1}{D_1} = \frac{L_1}{D_1}$, given $0 < \theta < 1, 0 < k < 1$and $0 < \delta < 1$, we have $\frac{\partial^2 L_1}{\partial {e_0}^2} > 0, \frac{\partial L_1(e_0)}{\partial e_0} > 0$. Thus, $L_1 > 0$ when $0 < \theta < 1, 0 < k < 1$and $0 < \delta < 1$ which implies that $\pi_m^{EU} > \pi_m^{NU}$ is established. Therefore, Part (i) of Proposition 1 holds.

Proposition 2 Subtracting π_m^{NR} from π_m^{ER} yields $\pi_m^{ER} - \pi_m^{NR} = \frac{A_2 {e_0}^2 - B_2 e_0 + C_2}{D_2} = \frac{L_2}{D_2}$. It is easy to verify $A_2 > 0$ and $D_2 > 0$ always exist. For $\pi_m^{ER} - \pi_m^{NR} = 0$, there are two root ${e_0}^{\#1} = \frac{B_2 - \sqrt{{B_2}^2 - 4A_2 C_2}}{2A_2}$; ${e_0}^{\#2} = \frac{B_2 + \sqrt{{B_2}^2 - 4A_2 C_2}}{2A_2}$; thus in the interval of ${e_0}^{\#1} < e_0 < {e_0}^{\#2}$, $\pi_m^{ER} - \pi_m^{NR} < 0$, when ${e_0}^{\#1} > e_0$ or ${e_0}^{\#2} < e_0$, $\pi_m^{ER} - \pi_m^{NR} > 0$ which implies that $\pi_m^{ER} > \pi_m^{NR}$ is established.

Proposition 3 Subtracting π_m^{NU} from π_m^{NR} yields $\pi_m^{NR} - \pi_m^{NU} = p_e S + \frac{(1 - e_0 p_e)^2 t}{2(4t - (p_e + \eta)^2)} - \frac{t}{8t - 2\eta^2} = L_3$, we have $\frac{\partial L_3}{\partial e_0} = \frac{p_e(1 - e_0 p_e)t}{-4t + (p_e + \eta)^2}$, and $\frac{\partial^2 L_3}{\partial {e_0}^2} = \frac{{p_e}^2 t}{4t - (p_e + \eta)^2} > 0$, When $p_e > \frac{1}{e_0}$, $\frac{\partial L_3}{\partial e_0} > 0$ and when $0 < p_e < \frac{1}{e_0}$, $\frac{\partial L_3}{\partial e_0} < 0$. Thus when the $L_3\left(\frac{1}{p_e}\right) \geq 0$, that is $S \geq S_1 = \frac{t}{8p_e t - 2p_e \eta^2}$, the government carbon quota is enough, in this case, for $\forall e_0 > 0$, $L_3 \geq 0$ exists, and $\pi_m^{NR} \geq \pi_m^{NU}$.

When $L_3\left(\frac{1}{p_e}\right) < 0$, by $L_3 = 0$ we can get two root, ${e_0}^{\#1}$ and ${e_0}^{\#2}$, now we should compare with ${e_0}^{*1}$, ${e_0}^{*2}$ and $\frac{1}{p_e}$, respectively. When $0 < p_e < \frac{1}{e_0}$, if $e_0 \in ({e_0}^{*1}, \frac{1}{p_e})$, $L_3 < 0$, $\pi_m^{NR} < \pi_m^{NU}$, if $e_0 \in (0, {e_0}^{*1})$, $L_3 > 0$, $\pi_m^{NR} > \pi_m^{NU}$. Similar, when $p_e > \frac{1}{e_0}$, if $e_0 \in (\frac{1}{p_e}, {e_0}^{*2})$, $L_3 < 0$, $\pi_m^{NR} < \pi_m^{NU}$, if $e_0 \in (\frac{1}{p_e}, {e_0}^{*2})$, $L_3 < 0$, $\pi_m^{NR} < \pi_m^{NU}$; finally, if $e_0 > {e_0}^{*2}$, $L_3 > 0$, $\pi_m^{NR} > \pi_m^{NU}$.

In conclusion, when the manufacturer decides not to encroach, if $S \geq S_1 = \frac{t}{8p_e t - 2p_e \eta^2}$, $\pi_m^{NR} \geq \pi_m^{NU}$. Otherwise, there exist the interval of e_0 that if $e_0 \in ({e_0}^{*1}, {e_0}^{*2})$, $\pi_m^{NR} < \pi_m^{NU}$; otherwise, $\pi_m^{NR} > \pi_m^{NU}$. In this proposition, ${e_0}^{*1} = \frac{(4t - (p_e + \eta)^2)(\frac{p_e t}{4t - (p_e + \eta)^2} - \sqrt{\frac{{p_e}^2 t(t - 8p_e S t + 2p_e S \eta^2)}{(4t - \eta^2)(4t - (p_e + \eta)^2)}})}{{p_e}^2 t}$; ${e_0}^{*2} = \frac{(4t - (p_e + \eta)^2)(\sqrt{\frac{{p_e}^2 t(t - 8p_e S t + 2p_e S \eta^2)}{(4t - \eta^2)(4t - (p_e + \eta)^2)}} + \frac{p_e t}{4t - (p_e + \eta)^2})}{{p_e}^2 t}$.

Proposition 4 Subtracting π_m^{EU} from π_m^{ER} yields $\pi_m^{ER}-\pi_m^{EU} = L_4$, $L_4 = ae_0{}^2 + be_0 + c$,

we have $\frac{\partial L_4}{\partial e_0} = \Big(p_e\Big(2(1-\delta)\big((1-\delta)\delta\eta^2(2-k\theta)^2(1-k\theta) + kt\theta(16-20\delta-26k\theta+4k(9-\delta)\delta\theta+k^2(4-\delta)(3-4\delta)\theta^2-k^3(2-\delta)(1-\delta)\theta^3)\big) + p_e\Big(2e_0(1-\delta)\delta^2\eta^2(2-k\theta)^2 + 2(1-\delta)\delta\eta(2-k\theta)^2(1-k(1-\delta)\theta) - 4e_0t(1-k\theta)\big(8-k\theta(5-k\theta)-\delta(4-k\theta)(2-k\theta)\big)\Big)\Big)\Big)/$
$(4p_e{}^2(1-k\theta)(8-k\theta(5-k\theta)-\delta(4-k\theta)(2-k\theta)) + 2(-1+\delta)(2(-1+\delta)\eta^2(1-k\theta)(8(1-\delta)-k(5-6\delta)\theta+k^2(1-\delta)\theta^2) + kt\theta(32(1-\delta)-4k(13-\delta)(1-\delta)\theta+4k^2(6-\delta)(1-\delta)\theta^2-k^3(2-\delta)^2\theta^3)) + 4P(-1+\delta)\eta(k\delta^2\theta(2-k\theta)^2+2\delta(4-k\theta)(2-k\theta)(1-k\theta)-2(1-k\theta)(8-k\theta(5-k\theta))))$, and $\frac{\partial^2 L_4}{\partial e_0{}^2} = (p_e{}^2(2(1-\delta)\delta^2\eta^2(2-k\theta)^2 - 4t(1-k\theta)(8-k\theta(5-k\theta)-\delta(4-k\theta)(2-k\theta))))/(4p_e{}^2(1-k\theta)(8-k\theta(5-k\theta)-\delta(4-k\theta)(2-k\theta)) - 2(1-\delta)(2(1-\delta)\eta^2(1-k\theta)(8(-1+\delta)+k(5-6\delta)\theta-k^2(1-\delta)\theta^2) + kt\theta(32(1-\delta)-4k(13-\delta)(1-\delta)\theta+4k^2(6-\delta)(1-\delta)\theta^2-k^3(2-\delta)^2\theta^3)) - 4p_e(1-\delta)\eta(k\delta^2\theta(2-k\theta)^2+2\delta(4-k\theta)(2-k\theta)(1-k\theta)-2(1-k\theta)(8-k\theta(5-k\theta))))$, in this case, $\frac{\partial^2 L_4}{\partial e_0{}^2} = 2a$, $\frac{\partial L_4}{\partial e_0} = 2ae_0 + b$, thus by $\frac{\partial L_4}{\partial e_0} - \frac{\partial^2 L_4}{\partial e_0{}^2} * e_0 = b$, put $-\frac{\frac{\partial L_4}{\partial e_0} - \frac{\partial^2 L_4}{\partial e_0{}^2} * e_0}{\frac{\partial^2 L_4}{\partial e_0{}^2}} = -\frac{b}{2a}$ into $L_4 = 0$ by Mathematica 13.0, we can get $L_4\left(-\frac{b}{2a}\right) = 0$, $S = S_2$, similar to proposition 2, when the $S \geq S_2$, government carbon quota is enough, and for $\forall e_0 > 0$, $L_3 \geq 0$ exists, we can get $\pi_m^{ER} \geq \pi_m^{NR}$.

When $L_4\left(-\frac{b}{2a}\right) < 0$, by $L_4 = 0$ we can get two root, $e_0{}^{*3}$ and $e_0{}^{*4}$, now we should compare $L_4(e_0{}^{*3})$, $L_4(e_0{}^{*4})$ and $L_4(-\frac{b}{2a})$ with 0, respectively. When $e_0 < -\frac{b}{2a}$, if $e_0 \in (e_0{}^{*3}, -\frac{b}{2a})$, $L_4 < 0$, $\pi_m^{NR} < \pi_m^{NU}$, if $e_0 \in (0, e_0{}^{*3})$, $L_4 > 0$, $\pi_m^{NR} > \pi_m^{NU}$. Similar, when $e_0 \geq -\frac{b}{2a}$, if $e_0 \in (-\frac{b}{2a}, e_0{}^{*4})$, $L_4 < 0$, $\pi_m^{ER} < \pi_m^{EU}$, if $e_0 \in (-\frac{b}{2a}, e_0{}^{*4})$, $L_4 < 0$, $\pi_m^{ER} < \pi_m^{EU}$; finally, if $e_0 > e_0{}^{*4}$, $L_4 > 0$, $\pi_m^{NR} > \pi_m^{NU}$.

In conclusion, when the manufacturer decides to encroach, if $S \geq S_2$, $\pi_m^{ER} \geq \pi_m^{EU}$. Otherwise, there exist the interval of e_0 that if $e_0 \in (e_0{}^{*3}, e_0{}^{*4})$, $\pi_m^{ER} < \pi_m^{EU}$; otherwise, $\pi_m^{ER} \geq \pi_m^{EU}$.

$t_1 = \frac{(1-\delta)\eta^2(1-k\theta)}{k\theta(2-k(2-\delta)\theta)}$;$t_2 = \frac{(P+\eta-\delta\eta)(P+\eta-\delta\eta+jP\delta\eta-k(1-\delta)(P+\eta)\theta)}{k\theta(2-2\delta+jP\delta-k(2-\delta)(1-\delta)\theta)}$;

$$t_3 = \frac{(-2+k\theta)(3P-2P\delta+3\eta-3\delta\eta-k(1-\delta)(P+\eta)\theta)(P+\eta-\delta\eta+jP\delta\eta-k(1-\delta)(P+\eta)\theta)}{2k(1-\delta)\theta(2-2k\theta-\delta(2-k\theta)(3-2k\theta))+jP(k\delta^2\theta(2-k\theta)^2-2(1-k\theta)(8-k\theta(5-k\theta))+\delta(2-k\theta)(8-k\theta(13-3k\theta)))}.$$

$$A_1 = k^2\theta^2(4(1-\delta)(5-9\delta)-4k(1-\delta)(6-13\delta)\theta+k^2(4-(20-17\delta)\delta)\theta^2),$$

$$B_1 = 2k(1-\delta)\eta^2\theta(1-k\theta)(7-10\delta-k(9-13\delta)\theta+2k^2(1-2\delta)\theta^2),$$

$$C_1 = (1-\delta)^2\eta^4(2-k\theta)^2(1-k\theta)^2,$$

$$D_1 = 2(4t-\eta^2)(kt\theta(32(1-\delta)-4k(13-\delta)(1-\delta)\theta+4k^2(6-\delta)(1-\delta)\theta^2-k^3(2-\delta)^2\theta^3)-2(1-\delta)\eta^2(1-k\theta)(8-k\theta(5-k\theta)-\delta(4-k\theta)(2-k\theta))).$$

$$A_2 = {P_e}^2(((1-\delta)\delta^2\eta^2(P_e+\eta)^2(2-\theta k)^2)-2t\delta\eta(16(1-\delta)(P_e+\eta)-(P_e(26-4(8-\delta)\delta)+(26-33\delta+6\delta^2)\eta)\theta k+(2P(6-\delta(9-2\delta))+(12-\delta(18-5\delta))\eta)(\theta k)^2-(2-\delta)(1-\delta)(P+\eta)(\theta k)^3)+t^2(64(1-\delta)-8(17-22\delta+4\delta^2)\theta k+4(25-\delta(41-(15-\delta)\delta))(\theta k)^2-4(1-\delta)(8-(7-\delta)\delta)(\theta k)^3+(2-\delta)^2(1-\delta)(\theta k)^4));$$

$$B_2 = 2P_e({P_e}^3(1-\delta)\delta\eta(2-\theta k)^2(1-(1-\delta)\theta k)+{P_e}^2((1-\delta)\delta\eta^2(2-\theta k)^2(3-\delta-3(1-\delta)\theta k)+t(1-(1-\delta)\theta k)(\delta^2(2-\theta k)^2\theta k-2(1-\theta k)(8-(5-\theta k)\theta k)+\delta(2-\theta k)(8-3(4-\theta k)\theta k)))+P_e(1-\delta)\eta(\delta\eta^2(2-\theta k)^2(3-2\delta-3(1-\delta)\theta k)-2t(\delta^2(2-\theta k)^2\theta k(1+\theta k)+2(1-\theta k)^2(8-(5-\theta k)\theta k)-\delta(2-\theta k)(1-\theta k)(4-\theta k(10-3\theta k))))+(1-\delta)((-1+\delta)\delta\eta^4(2-\theta k)^2(-1+\theta k)+t^2\theta k(-8\delta(-3+\theta k)(2-\theta k)(1-\theta k)+3\delta^2(2-\theta k)^2\theta k-4(1-\theta k)(8-(5-\theta k)\theta k))-t\eta^2(2(1-\theta k)^2(8-(5-\theta k)\theta k)+\delta^2(2-\theta k)\theta k(2-(\theta k)^2)-\delta(1-\theta k)(16-\theta k(26-\theta k(16-3\theta k))))));$$

$$C_2 = {P_e}^4(1-\delta)(2-\theta k)^2(1-(1-\delta)\theta k)^2+2{P_e}^3(1-\delta)\eta(2-\theta k)^2(1-(1-\delta)\theta k)(2-\delta-2(1-\delta)\theta k)+(1-\delta)((1-\delta)^2\eta^4(2-3\theta k+(\theta k)^2)^2+2t(1-\delta)\eta^2(1-\theta k)\theta k(7-(9-2\theta k)\theta k-\delta(2-\theta k)(5-4\theta k))+t^2(\theta k)^2(4(5-\theta k)(1-\theta k)-4\delta(1-\theta k)(14-5\theta k)+\delta^2(2-\theta k)(18-17\theta k)))+2P_e(-1+\delta)\eta(-((-1+\delta)\eta^2(2-\theta k)^2(-1+\theta k)(2-\delta-2(1-\delta)\theta k))-t\theta k(2(1-\theta k)^2(7-2\theta k)-2\delta(1-\theta k)(14-3\theta k(7-2\theta k))+\delta^2(2-\theta k)(6-\theta k(15-8\theta k))))+{P_e}^2(((1-\delta)\eta^2(2-\theta k)^2(6-6\delta+\delta^2-6(2-\delta)(1-\delta)\theta k+6(1-\delta)^2(\theta k)^2))+2t\theta k(1-(1-\delta)\theta k)(7-9\theta k+2((\theta k)^2+\delta^2(2-\theta k)(3-2\theta k)-\delta(9-(11-3\theta k)\theta k))));$$

$$D_2 = {P_e}^2(((1-\delta)\delta^2\eta^2(P_e+\eta)^2(2-\theta k)^2)-2t\delta\eta(16(1-\delta)(P_e+\eta)-(P_e(26-4(8-\delta)\delta)+(26-33\delta+6\delta^2)\eta)\theta k+(2P_e(6-\delta(9-2\delta))+(12-\delta(18-5\delta))\eta)(\theta k)^2-(2-\delta)(1-\delta)(P+\eta)(\theta k)^3)+t^2(64(1-\delta)-8(17-22\delta+4\delta^2)\theta k+4(25-\delta(41-(15-\delta)\delta))(\theta k)^2-4(1-\delta)(8-(7-\delta)\delta)(\theta k)^3+(2-\delta)^2(1-\delta)(\theta k)^4));$$
